# Bacterial vaginosis toxins impair sperm capacitation and fertilization

**DOI:** 10.1093/humrep/deaf132

**Published:** 2025-09-01

**Authors:** Shweta Bhagwat, Leila Asadi, Ronald McCarthy, Juan Ferreira, Ping Li, Ethan Li, Sariela Spivak, Ariana Gaydon, Vaka Reddy, Christy Armstrong, Sydney R. Morrill, Hillary Zhou, Amanda L. Lewis, Warren G. Lewis, Celia M. Santi

**Affiliations:** 1 Department of Obstetrics and Gynecology, Washington University School of Medicine, Saint Louis, MO, USA; 2 Department of Obstetrics, Gynecology, and Reproductive Sciences, Glycobiology Research and Training Center, University of California San Diego, La Jolla, CA, USA

**Keywords:** sperm capacitation, hyperactivation, acrosomal exocytosis, infertility, bacterial vaginosis, lipopolysaccharide, vaginolysin, CatSper, toll-like receptor 4

## Abstract

**STUDY QUESTION:**

What effect do toxins produced by bacterial vaginosis (BV) bacteria have on sperm function?

**SUMMARY ANSWER:**

BV toxins dysregulate sperm capacitation and intracellular calcium homeostasis, and impair the ability of sperm to fertilize oocytes.

**WHAT IS KNOWN ALREADY:**

In BV, which is linked to infertility, overgrowth of *Prevotella* and *Gardnerella* in the vagina is accompanied by elevated concentrations of the toxins lipopolysaccharide (LPS) and vaginolysin (VLY).

**STUDY DESIGN, SIZE, DURATION:**

This was a laboratory study in which human semen samples were collected from consenting healthy donors with normal semen parameters. Mouse sperm samples were obtained from the caudal epididymis.

**PARTICIPANTS/MATERIALS, SETTING, METHODS:**

Motile mouse and human sperm were isolated via swim-up and treated under non-capacitating or capacitating conditions. LPS from *Escherichia coli* was commercially available. VLY was produced by cloning the *Gardnerella* VLY protein in the ClearColi expression system. Mouse sperm were pre-incubated in IVF medium with LPS or VLY and then co-cultured with ovulated cumulus–oocyte complexes. The effects of LPS and VLY on sperm motility and hyperactivation were assessed with computer-assisted sperm analysis. Effects on viability were assessed by Hoechst staining. Acrosomal exocytosis was assessed in sperm from transgenic Acr-eGFP mice and in human sperm stained with *Pisum sativum* agglutinin FITC. Intracellular calcium concentration was measured by using the calcium-sensitive dye Fluo-4 AM and fluorescence microscopy. The effects of LPS on sperm from CatSper knockout mice were assessed. Additionally, sperm were treated with a Toll-like receptor 4 (TLR4) antagonist and further exposed to LPS.

**MAIN RESULTS AND THE ROLE OF CHANCE:**

Exposure of mouse sperm to LPS or VLY significantly decreased IVF (*P* < 0.05). Under capacitating conditions, both toxins initially increased mouse (*P* < 0.001) and human (*P* < 0.05) sperm hyperactivation, then significantly decreased sperm motility (*P* < 0.05), hyperactivation (*P* < 0.05), and acrosomal exocytosis (*P* < 0.01). These changes were accompanied by a rapid and irreversible increase in sperm intracellular calcium concentration. Effects of LPS, but not VLY, were prevented by polymyxin B, which binds LPS. The LPS-induced intracellular calcium increase required external calcium, but not the calcium channel CatSper, and was inhibited by a TLR4 antagonist.

**LARGE SCALE DATA:**

N/A.

**LIMITATIONS, REASONS FOR CAUTION:**

First, the commercially available LPS we used was isolated from *Escherichia coli*, rather than from the BV-associated bacteria *Prevotella bivia*. Second, we did not quantify the absolute sperm intracellular calcium concentration before or after LPS or VLY treatment. Third, all of our experiments were *in vitro*.

**WIDER IMPLICATIONS OF THE FINDINGS:**

These studies suggest that BV-associated toxins contribute to infertility, in part, by impairing sperm capacitation and reducing their fertilizing ability.

**STUDY FUNDING/COMPETING INTEREST(S):**

This work was supported by the National Institutes of Health (grant number R01 HD069631 to C.M.S.). The authors declare that they have no conflict of interest.

## Introduction

Bacterial vaginosis (BV) is characterized by a disruption of the vaginal microbiota, with a decrease in protective *Lactobacillus* species and overgrowth of anaerobic bacteria, including *Gardnerella* and *Prevotella* (Ravel et al., 2011). BV affects ~29% of reproductive-aged women in the USA (Allsworth and Peipert, 2007), and is associated with various adverse health outcomes, such as sexually transmitted infections, pelvic inflammatory disease (Ravel et al., 2021; Turpin et al., 2021), and preterm birth (Salminen et al., 2008). Moreover, BV has been linked to female infertility through several mechanisms (Ravel et al., 2021; Cocomazzi et al., 2023). First, elevated concentrations of proinflammatory cytokines and chronic inflammation in the reproductive tract lead to tissue damage and impaired implantation (Murphy and Mitchell, 2016; Ravel et al., 2021). Second, BV-associated bacteria and their metabolites induce endometrial dysfunction (Łaniewski and Herbst-Kralovetz, 2021), interfere with embryo implantation, and cause early pregnancy loss (Eckert et al., 2003; van Oostrum et al., 2013; Haahr et al., 2019; Hong et al., 2020). Third, imbalances in vaginal pH and microbiota (Amsel et al., 1983; Mania-Pramanik et al., 2008), along with the toxins produced by *Gardnerella* and *Prevotella*, adversely affect sperm quality and motility (Li et al., 2016; O’Doherty et al., 2016).

*Prevotella* is an opportunistic pathogen in the female genital tract (George et al., 2024). Like other *Proteobacteria*, it produces lipopolysaccharide (LPS) (Aroutcheva et al., 2008), a heat-stable toxin (also known as endotoxin). LPS reaches two to three orders of magnitude higher concentrations in the vagina of those with BV than in those without BV. These higher LPS concentrations strongly correlate with the abundance of *Prevotella* in BV-positive individuals (Aroutcheva et al., 2008). LPS-mediated inflammation has been linked with female infertility, affecting ovarian and pituitary function (Magata et al., 2023). Additionally, in several animal models, a LPS challenge can trigger preterm birth and injure the fetal brain (Wang et al., 2006; Firmal et al., 2020). Unlike *Prevotella*, *Gardnerella* does not make LPS but instead produces the toxin vaginolysin (VLY). VLY is a cholesterol-dependent cytolysin that forms pores in human cell membranes and is active against erythrocytes, as well as vaginal and cervical cells *in vitro* (Yoo and Lee, 2016; Ragaliauskas et al., 2019; Campisciano et al., 2021).

We hypothesize that LPS and VLY contribute to female infertility by disrupting sperm capacitation, which occurs in the female genital tract and is necessary for a sperm to be able to fertilize an oocyte. One component of capacitation is hyperactivation, which causes flagellar beating to increase in amplitude, decrease in frequency, and become asymmetrical (Suarez, 2008). Capacitation also involves acrosomal exocytosis (Yanagimachi, 1994; Molina et al., 2018), in which the outer acrosomal membrane at the sperm head fuses with the sperm plasma membrane to release the acrosomal content in response to a stimulus (Sosa et al., 2015). Hyperactivation and acrosomal exocytosis are necessary for sperm to penetrate the cumulus and zona pellucida layers surrounding the oocyte and fuse with the oocyte.

Here, we show that both LPS and VLY caused mouse and human sperm to hyperactivate early during capacitation. Prolonged exposure to LPS and VLY decreased sperm motility, hyperactivation, and fertilization capability. Furthermore, sperm acrosomal exocytosis was inhibited by the two toxins. In defining the underlying mechanisms, we found that LPS and VLY caused rapid and irreversible increases in intracellular calcium (Ca^2+^) concentration in sperm. Although hyperactivation and acrosomal exocytosis require an increase in intracellular Ca^2+^, an excessive intracellular Ca^2+^ concentration in mammalian sperm impairs mitochondrial function, leading to a loss of motility, activation of apoptotic cascades, and premature loss of the acrosome (Liu and Baker, 1990; Zhu et al., 2016). Finally, we show that the LPS-induced Ca^2+^ entry involved Toll-like receptor 4 (TLR4). VLY-induced Ca^2+^ entry is likely due to the membrane pores formed by this toxin. Together, our data show that toxins present in BV dysregulate capacitation and intracellular Ca^2+^ homeostasis, impairing the ability of sperm to fertilize oocytes. This mechanism may contribute to infertility in patients with BV.

## Materials and methods

### Reagents

All reagents used to prepare human tubal fluid (HTF) and Toyoda–Yokoyama–Hosi (TYH) media, as well as the Ca^2+^ ionophore A23187, polymyxin B (PMB), TAK-242 (TLR4 receptor inhibitor), progesterone, Methyl-ß-cyclodextrin, and immunoglobulin-free bovine serum albumin (BSA) were from Millipore Sigma (St Louis, MO, USA). Dulbecco’s phosphate-buffered saline (DPBS) tissue culture grade was from GIBCO Life Technologies (Gaithersburg, MD, USA); Hoechst 33342 was from Cayman Chemicals (Ann Arbor, MI, USA); Fluo-4 AM was from Invitrogen^™^ Thermo Fisher Scientific (Waltham, MA, USA); paraformaldehyde was from Electron Microscopy Sciences (Hatfield, PA, USA); and LPS from *E. coli* strain O112: H10 and the Limulus amebocyte lysate chromogenic assay were from Associates of Cape Cod Inc. (E. Falmouth, MA, USA).

### Animals and ethics statement

All mouse procedures were performed according to the National Institutes of Health Guiding Principles for the care and use of laboratory animals. These procedures were reviewed and approved by the Institutional Animal Care and Use Committee of Washington University in St Louis (St Louis, MO, USA) (protocol number 20-0126).

C57BL/6 and CatSper1 knockout male mice (CatSper KO) (60–90days old) were purchased from Jackson Labs and kept at a constant temperature of 22 ± 2°C under a 12/12h dark/light cycle with free access to food and water. Oocytes for IVF experiments were obtained from 4- to 8-week-old C57BL/6 female mice.

### Human participants

This study conformed with the Declaration of Helsinki (except for registration in a database) and was approved by the Washington University in St Louis Institutional Review Board (protocol number 201706077). All human participants signed written informed consent forms approved by the Washington University in St Louis Institutional Review Board.

### Mouse sperm collection and capacitation

Sexually mature male mice (60–90days old) were euthanized via cervical dislocation, and sperm were isolated from the cauda epididymis. Sperm were allowed to swim-up in non-capacitating (NC) TYH media buffered with HEPES (in mM: 119.3 NaCl, 4.7 KCl, 1.71 CaCl_2_.2H_2_O, 1.2 KH_2_PO_4_, 1.2 MgSO_4_.7H_2_O, 5.56 glucose, 0.51 sodium pyruvate, 10 HEPES) at pH 7.4 for 15–20 min at 37°C, unless specified. The motile fraction of the sample was removed from the tube. To induce capacitation, sperm were incubated in capacitating (CAP) TYH medium supplemented with 5mg/ml BSA and 15mM NaHCO_3_ at 37°C for 60–90 min. NC sperm were incubated in TYH without BSA and NaHCO_3_ unless specified otherwise.

### Human sperm preparation

Human semen samples were obtained by masturbation after 3–5days of abstinence from the Washington University Fertility and Reproductive Medicine Center. The clinic confirmed that all samples met normal semen parameters according to the World Health Organization (≥ 15 million sperm per ml, ≥ 40% total motility, ≥ 32% progressive motility). Samples were allowed to liquefy for 1h at room temperature, and then sperm were purified by swim-up as previously described (Molina et al., 2020). Briefly, within 2h of production, sperm were allowed to swim up in non-commercial HTF (in mM: 98 NaCl, 4.7 KCl, 0.4 KH_2_PO_4_, 2 CaCl_2_, 0.2 MgSO_4_, 20 HEPES, 3 glucose, 21 lactic acid, 0.3 sodium pyruvate; pH adjusted to 7.4 with NaOH) for 1h at 37°C without CO_2_. To induce capacitation, sperm were incubated in CAP HTF medium supplemented with 5mg/ml BSA and 25mM NaHCO_3_ at 37°C for a maximum of 180 min.

### Production of *Gardnerella* VLY protein

A truncated mutant *vly* gene from *Gardnerella* ATCC14019 (Randis et al., 2009) was cloned into a pET28a vector, which was transformed into ClearColi BL21(DE3) cells (Biosearch Technologies, Teddington, UK) with detoxified LPS. Protein was expressed and purified as previously described (Morrill et al., 2023). Cytolytic activity on human red blood cells was verified by serial dilution and assay of hemoglobin release measured at 545nm with an EC50 of ~10ng/ml (Supplementary Fig. S1) as previously described (Morrill et al., 2023). In parallel, an empty vector was used to prepare a mock expression and purification that lacked VLY but contained all other reagents. VLY and mock preparations were stored in 0.5× phosphate-buffered saline (PBS) 20% glycerol at −20°C.

### LPS precautions and testing

LPS from Associates of Cape Cod, Inc. (E Falmouth, MA) was dissolved according to the manufacturer’s instructions. Dilutions were performed carefully to minimize LPS loss, avoiding repeated transfers in plastic to prevent adsorption. Because LPS can contaminate reagents, especially recombinant proteins, pyrogen-free reagents, and labware were used when available. Clean glassware was dry-baked at 200C for 2h to destroy LPS. The LPS content of reagents was quantified by using a chromogenic Limulus Amoebocyte Lysate assay from Associates of Cape Cod (Supplementary Table S1) according to the manufacturer’s instructions. Protein preparations and buffers (TYH and HTF) contained < 1 EU/ml (approximately < 0.1ng/ml) of LPS at the concentrations used in sperm preparation and assays.

### IVF protocol

Sperm from C57BL/6 male mice (4–8weeks old) were prepared as described above and capacitated for 3h in TYH-CO_2_ buffered media supplemented either with 0.75mM Methyl-ß-cyclodextrin (Nakagata, 2011) or 5mg/ml BSA at 37°C and 5% CO_2_ with or without LPS or VLY. C57BL/6 female mice (4–8weeks old) were superovulated by intraperitoneally injecting 5–7.5IU pregnant mare serum gonadotropin (ProSpec Cat No. HOR-272). After 48h, these mice were injected with 5–7.5IU human chorionic gonadotropin (Millipore/Sigma CG5–1VL). Twelve to fifteen hours later (at the 3-h sperm capacitation timepoint), female mice were euthanized, and oocyte–cumulus complexes were isolated in 200 μl of high Ca^2+^ HTF (Nakagata, 2011). The media in the dish overlayed with Ovoil was allowed to equilibrate for at least 1h in an incubator at 37°C and 5% CO_2_, before the addition of oocytes. Fertilization wells containing 20–30 oocytes were mixed with CAP sperm (final concentration of 2 × 10^6^ sperm/ml) and incubated for 4–6h. Oocytes were then washed three times and placed in a drop of HTF medium with a disposable soda-lime glass 50 μl microcapillary pipette (Kimble Cat No. 71900-50) attached to an aspirator tube assembly (Millipore/Sigma A5177–5EA). Oocytes were counted, and the dish was incubated overnight at 37°C and 5% CO_2_. The percentage of fertilized oocytes was calculated by dividing the number of two-cell embryos by the total number of oocytes.

### Computer-assisted sperm analysis

Mouse and human sperm were suspended in their respective CAP media and exposed to 1 μg/ml LPS or VLY (mouse sperm) and 0.1 μg/ml LPS or VLY (human sperm) from the start of capacitation. Sperm total and hyperactivated motility were measured at time 0 and at 0.5–1h intervals. Sperm samples (3 μl) from each condition were placed into 20-micron Leja standard count four-chamber slides, pre-warmed at 37°C, and a minimum of 200 cells were counted. Computer-assisted sperm analysis (CASA) was performed with a Hamilton–Thorne digital image analyzer (HTR-CEROS II v.1.7; Hamilton-Thorne Research, Beverly, MA, USA). CASA settings were as previously described (Molina et al., 2020). For mouse sperm, hyperactivated motility was defined as curvilinear velocity (VCL) >150 μm/s, lateral head displacement (ALH) >7.0 μm, and linearity coefficient (LIN) of 32% at 60Hz (Mortimer et al., 1998). For human sperm, hyperactivated motility was defined as VCL ≥ 150 μm/s, ALH ≥ 7 μm, LIN ≤ 0% at 60Hz. Percent hyperactivation was calculated by dividing the number of hyperactivated sperm by the number of total motile sperm. Total and progressive motility values were obtained as percentages from the CASA software.

### Sperm viability assessment

Sperm viability was quantified with Hoechst 33342 (900nM, Cayman Chemical Company) (Lyon et al., 2023). After 2–5min, fluorescence intensity was measured with an Aurora 4l 16V-14B-10YG-8R Flow Cytometer and a V3 or 458 nm filter (Lyon et al., 2023). At least 10000 sperm were recorded for each sample. The fluorescence cutoff was selected by measuring the fluorescence of sperm killed by treatment with 3–6% sodium hypochlorite for 2–5min. Unstained and 3% bleach-treated cells were used as reference for minimal and maximal Hoechst fluorescence measurements (Supplementary Fig. S2).

### Sperm intracellular calcium concentration measurement

After sperm swim-up in NC or CAP conditions for 15–20 min, motile sperm were collected and incubated with 10 μM Fluo-4 AM and 0.05% Pluronic F-127 at 37°C for 60 min. Sperm were centrifuged at 300–400*g* for 5–10 min, resuspended in the corresponding media, and allowed to attach to poly-l-lysine (0.1%) coated coverslips for 5 min. A local perfusion device with an estimated exchange time of 10s was used to apply test solutions.

Sperm [Ca^2+^]_i_ was recorded at room temperature with a Leica AF 6000LX system connected to a Leica DMi8000 inverted microscope, equipped with a 63X objective (HC PL FluoTar L 63X/0.70 Dry) and an Andor-Zyla-VCS04494 camera. Illumination was provided by a halogen lamp with a 488 ± 20 nm excitation filter and a 530 ± 20 nm emission filter. Data acquisition was performed using Leica LAS X software. Acquisition parameters used were: 20 ms exposure time, 4 × 4 binning, 1024 × 1024 pixels resolution (Ferreira et al., 2021). For each condition, an initial baseline reading was followed by the addition of LPS or VLY and a final perfusion with 5 μM Ionomycin (Iono) at the end of every experiment. Whole images were collected every 10s. LAS X, ImageJ, Clampfit 10 (Molecular Devices), and GraphPad were used for data analysis. Fluorescence was collected from a region of interest located in the sperm head (Chávez et al., 2014). To compare fluorescence across different samples and experimental conditions, Fluo-4 fluorescence (F) in response to LPS or VLY for each sperm was normalized to its respective *F*_Iono_ (*F*/*F*_iono_), after background subtraction. Cells with [Ca^2+^]_i_ increases of >10% of that obtained with Iono were counted as responsive. Percentages of cells responding to LPS or VLY were calculated by dividing the number of cells responding to the respective toxin by the total number of cells that responded to Ionomycin (IONO). Clampfit 10 software was used to measure amplitude and Tau (time that takes for Fluo-4 fluorescence to reach 63% of its final value) in responding sperm (Ferreira et al., 2021).

### Acrosomal exocytosis

Acrosomal exocytosis in mouse sperm was assessed in both NC and capacitated (CAP) sperm from transgenic mice expressing green fluorescent protein (GFP) in the acrosome (Hasuwa et al., 2010). Acrosomal exocytosis was induced by incubating sperm with 15 μM Ca^2+^ ionophore A23187 or 50 mM KCl (Millipore Sigma, St Louis) for 30 min before the end of capacitation. LPS or VLY (1 μg/ml) was added to CAP and NC samples either since the beginning of capacitation or together with the inducers A23187 and KCl at the last 30 min of capacitation. Sperm from all conditions were fixed in 4% paraformaldehyde and centrifuged at 300–400 *g* for 5–10 min. Supernatants were removed, the sperm were washed with PBS, and 15 μl of each sample was smeared onto a glass slide. Slides were air-dried and coverslips were mounted with 1:1 glycerol: pyrogen-free DPBS at room temperature. Sperm were examined at 40 × on an EVOS FL Cell Imaging System epifluorescence microscope (Thermo Fischer Scientific). Sperm were classified as acrosome-reacted if the acrosomal region lacked GFP fluorescence, and as non-reacted if GFP fluorescence was retained. A minimum of 200 sperm were evaluated per experiment. Acrosomal exocytosis was expressed as the percentage of reacted sperm relative to the total sperm counted per sample.

Human sperm acrosomal exocytosis was measured as previously described (Lyon et al., 2023). Briefly, swim-up sperm were incubated in both NC and CAP media (HTF supplemented with 25 mM HCO_3_ and 5mg/ml BSA) for 3 h. Acrosomal exocytosis was induced by adding 10 μM Ca^2+^ ionophore A23187 or 10 μM progesterone for 30 min. LPS or VLY (0.1 μg/ml) was added immediately after swim-up or during acrosomal exocytosis induction. After induction, sperm were fixed with 3% paraformaldehyde in DPBS at 4°C, smeared on slides, and stained with FITC-labeled *Pisum sativum* agglutinin (Millipore Sigma). Fluorescence was imaged as described for mouse sperm acrosomal exocytosis. Sperm with bright, uniformly stained acrosomes were counted as intact, and sperm with equatorial or no staining in the acrosomal region were counted as reacted. The minimum number of sperm and method for calculating percent acrosomal exocytosis were as described for mouse sperm.

### Statistical analyses

GraphPad Prism version 10.1.0 for Windows (GraphPad Software, La Jolla, CA) was used for all statistical analyses. An unpaired two-tailed Student’s *t*-test (alpha < 0.05) was used to compare sperm samples from different individuals, whereas a paired two-tailed *t*-test was used to compare control and treatment in the same sample, unless otherwise indicated. For time series data analysis, where time and the treatment groups were the two variables, two-way ANOVA or mixed model with Bonferroni’s multiple comparison test was performed. Data are expressed as the mean ± SD. *P* < 0.05 was considered statistically significant.

For experiments with human sperm, power analyses were performed for key comparisons in human sperm, including control versus treated conditions for sperm hyperactivation, total motility, and progressive motility. The power estimate for this study was based on our preliminary data. In the original dataset, our sample sizes for LPS-treated conditions were: n = 5 for sperm hyperactivation, total motility, and progressive motility. For VLY, the corresponding sample sizes were n = 9. Using a two-sample comparison for continuous endpoints (CAP control vs CAP treated), we obtained a power of 0.8 (α = 0.05, β = 0.2). Based on this analysis, the required sample sizes for LPS were 5, 11, and 7 for sperm hyperactivation, total motility, and progressive motility, respectively. For VLY, the required sample sizes were 6, 12, and 11. Accordingly, the number of biological replicates for all three motility parameters was increased to n = 12 for both LPS and VLY.

## Results

### LPS and VLY interfere with mouse sperm fertility and capacitation

**Figure 1.**
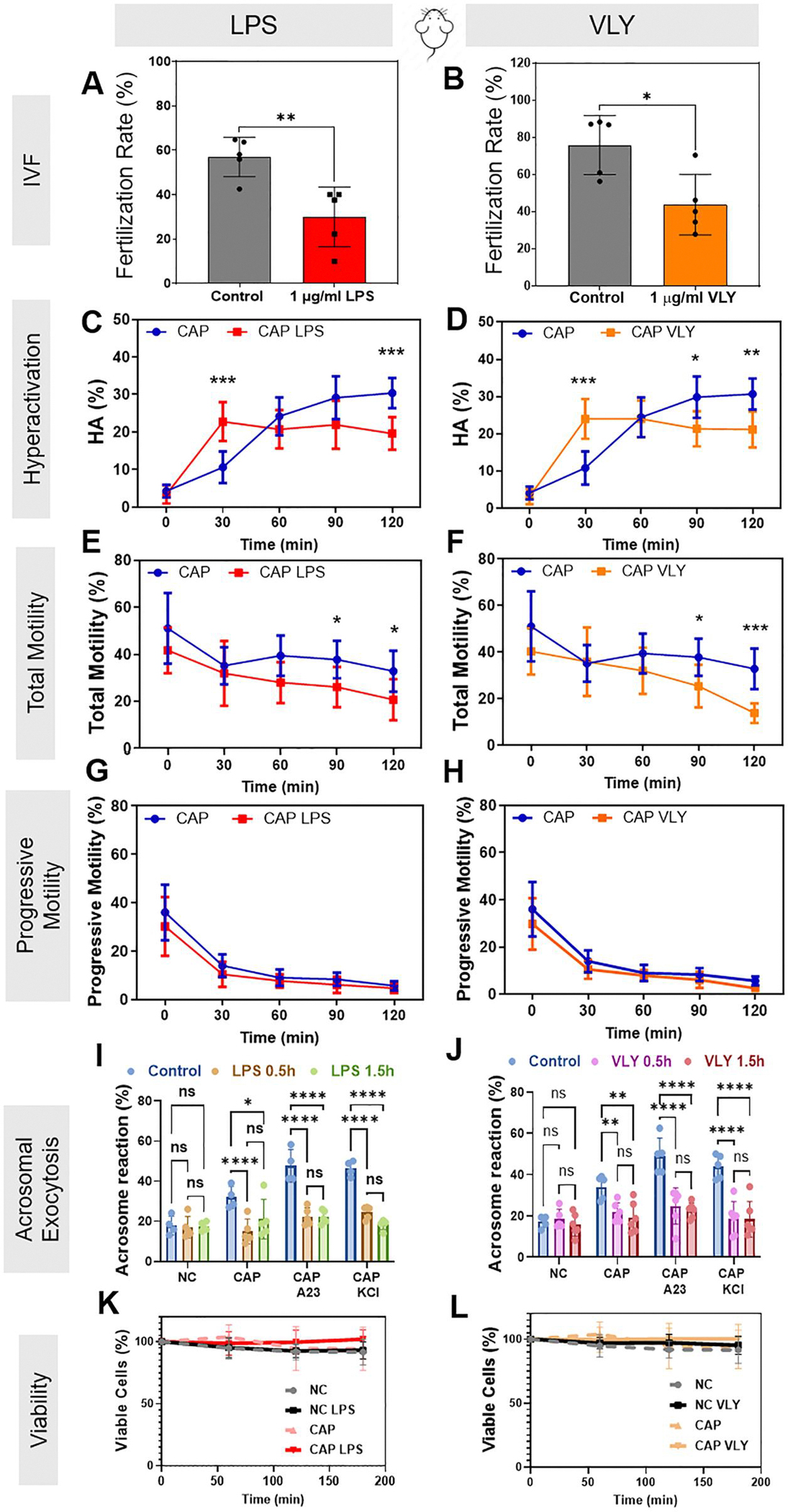
Lipopolysaccharide (LPS) and vaginolysin (VLY) impair mouse sperm fertility, hyperactivation, total motility, and acrosomal exocytosis in capacitating (CAP) conditions. Percentage of oocytes that reached the two-cell stage within 24 h after IVF using sperm previously treated for 3 h with 1 μg/ml (**A**) LPS (n = 5 biological replicates) or (**B**) VLY (n = 5 biological replicates) were calculated. CASA measurements were obtained for (**C, D**) hyperactivated motility (HA), (**E, F**) total motility, and (**G, H**) progressive motility of mouse sperm incubated under CAP conditions in the presence and absence of 1 μg/ml (**C, E, G**) LPS (n = 10 biological replicates) or (**D, F, H**) VLY (n = 9 biological replicates). Here, 0 min is the timepoint of bovine serum albumin (BSA)+sodium bicarbonate addition to initiate *in vitro* capacitation. Acrosomal exocytosis (AE) was quantified in non-capacitating (NC), and CAP sperm (200 each, from five biological replicates) incubated with (**I**) LPS or (**J**) VLY, for 0.5 and 1.5 h. Similarly, AE induced by 15 μM A23187 (A23) or 50 mM potassium chloride (KCl), was also quantified in CAP sperm. Sperm viability for NC and CAP sperm (n ≥ 3 biological replicates) was analyzed in the presence of 1 μg/ml (**K**) LPS or (**L**) VLY (10000 sperm each). Data are presented as mean and SD. *P < 0.05, **P < 0.01, *P* <0.001, *P* < 0.001, ns, non-significant. For (**A, B**), a paired *t*-test was used. For (**C–L**), two-way ANOVA with Bonferroni’s multiple comparison test was used.

To determine whether LPS or VLY impair the ability of sperm to fertilize oocytes, we capacitated mouse epididymal sperm in the presence and absence of 1 μg/ml LPS or 1 μg/ml VLY for 3 h. We then performed mouse IVF. When sperm were capacitated in the presence of LPS, the fertilization rate was 34.93 ± 8.57%, significantly lower than the rate of 60.57 ± 4.28% in control conditions. Similarly, fertilization rates decreased from 76 ± 16% to 44 ± 16% in the presence of VLY (Fig. 1A and B). The addition of LPS or VLY to the fertilization drop after capacitation had no effect on fertilization rates (53.76 ±2 5.43 for LPS, 71.26 ± 10.89 for VLY).

Next, we wanted to determine which outcomes of capacitation (hyperactivation, acrosomal exocytosis, or both) were affected by these toxins. Mouse sperm were incubated in CAP media with or without 1 μg/ml LPS or VLY, and motility was measured every 30 min for 120 min. At 30 min, the percentages of hyperactivated sperm were significantly higher in the presence of LPS or VLY than in the controls (Fig. 1C and D). However, at 90 and 120 min, sperm hyperactivation and total motility were significantly lower in the presence of LPS or VLY than in controls (Fig. 1C–F). No effect was observed on progressive motility in CAP (Fig. 1G and H) or in non-CAP conditions (Supplementary Fig. S3). These results suggest that LPS and VLY impair sperm hyperactivation, which is required for fertilization.

To measure the impact of LPS and VLY on acrosomal exocytosis, mouse sperm were exposed to 1 μg/ml LPS or VLY in CAP conditions, and acrosomal exocytosis was induced by adding 15 μM Ca^2+^ ionophore A23187 (A23) or 50 mM potassium chloride (KCl) (De La Vega-Beltran et al., 2012). As reported earlier by De La Vega-Beltran et al., about 40–60% of mouse sperm underwent induced acrosomal exocytosis in control CAP conditions. The presence of LPS or VLY significantly inhibited A23- and KCl-induced sperm acrosomal exocytosis but had no significant effect on acrosomal exocytosis in non-CAP conditions (Fig. 1I and J), (Liu and Baker, 1990).

**Figure 2.**
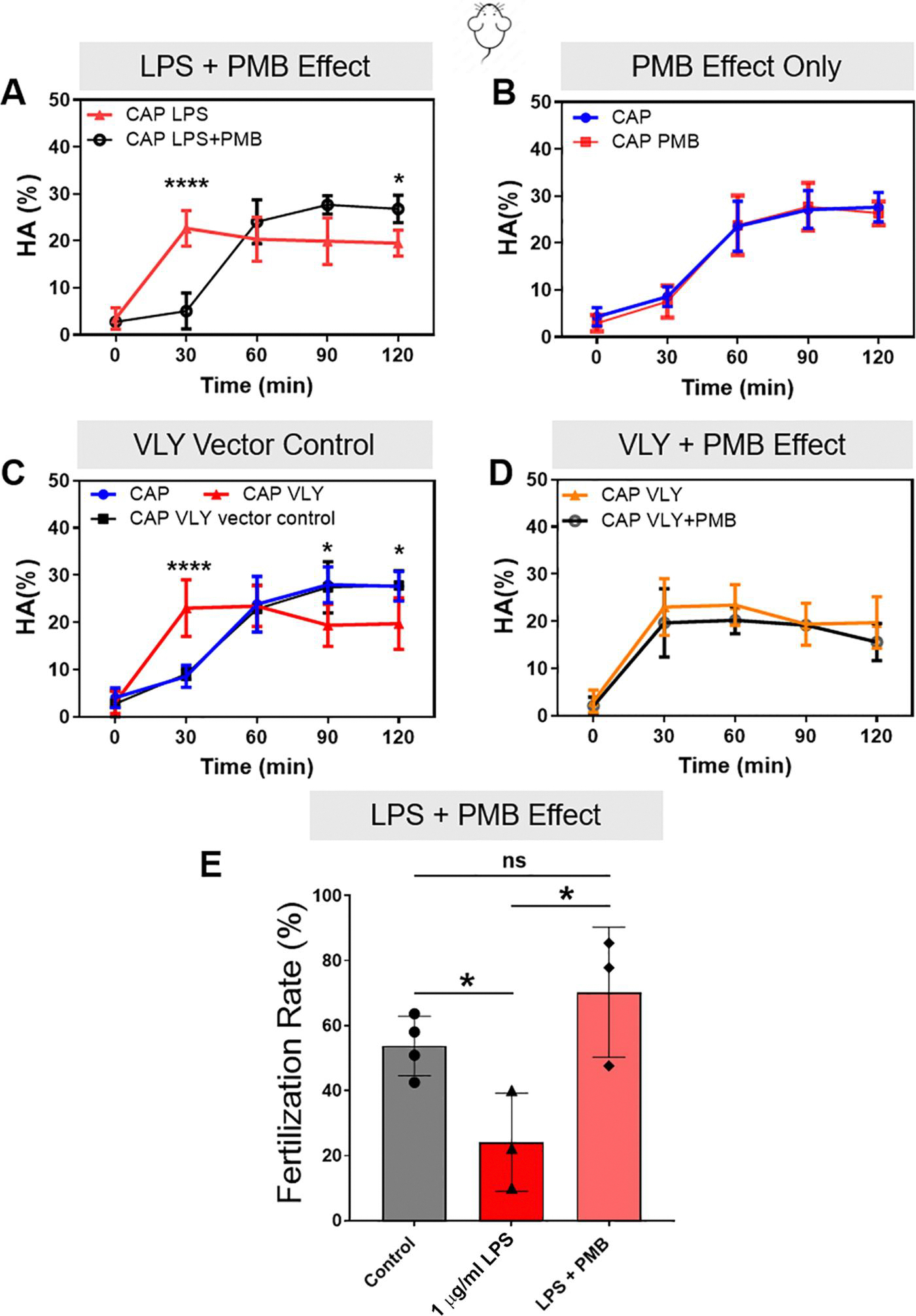
Effects of lipopolysaccharide (LPS) and vaginolysin (VLY) on mouse sperm are specific. CASA measurements were obtained for hyperactivated motility (HA) of mouse sperm incubated under capacitating (CAP) conditions in the presence of (**A**) 1 μg/ml LPS or 100 μg/ml polymyxin B (PMB)+1 μg/ml LPS, (**B**) CAP media or 100 μg/ml PMB alone, (**C**) CAP media, 1 μg/ml VLY, or VLY empty vector control, and (**D**) 1 μg/ml VLY or 100 μg/ml PMB+1 μg/ml VLY. Here, 0 min is the timepoint of bovine serum albumin (BSA)+sodium bicarbonate addition to initiate *in vitro* capacitation. (**E**) Percentage of oocytes that reached the two-cell stage within 24 h after IVF using sperm (n = 4 biological replicates) previously treated for 3 h without LPS, or with 1 μg/ml LPS or 100 μg/ml PMB+1 μg/ml LPS, were also calculated. Data are presented as mean and SD (n = 4 biological replicates for all experiments). *P < 0.05, ***P<0.0001, ns, non-significant. Data were analyzed by two-way ANOVA with Bonferroni’s multiple comparison test.

Next, we assessed whether these effects were specific to hyperactivation and acrosomal exocytosis or caused by a decrease in sperm viability. We found that mouse sperm viability did not significantly differ in the absence or presence of either toxin over 180 min (Fig. 1K and L). To test whether the effects were specifically due to LPS, we capacitated sperm in the presence of LPS plus PMB, which forms aggregates with LPS and hampers its activity. In this condition, LPS had no effect on mouse sperm hyperactivation (Fig. 2A) or IVF rates (Fig. 2E). PMB alone had no effect on hyperactivation (Fig. 2B), and it did not prevent the VLY-induced inhibition of hyperactivated motility (Fig. 2D). As a control for VLY specificity, we treated sperm with protein generated in parallel using an empty vector control. This treatment did not impair hyperactivation (Fig. 2C).

Because we added 15mM HCO_3_^−^ to the medium to induce capacitation, we wanted to determine whether the observed initial effects were due to changes in osmolarity. Therefore, we increased the external medium osmolarity by adding 15 mM NaCl instead of NaHCO_3_ (from 273.67 ± 1.53 to 304 ± 3.61 mOsm) and found that neither LPS nor VLY induced hyperactivated motility under this condition (Supplementary Fig. S4). Thus, we concluded that the effects on hyperactivation were due to changes in the capacitation signaling pathway induced by HCO_3_^−^ and not by changes in external osmolarity.

### LPS and VLY interfere with human sperm capacitation

**Figure 3.**
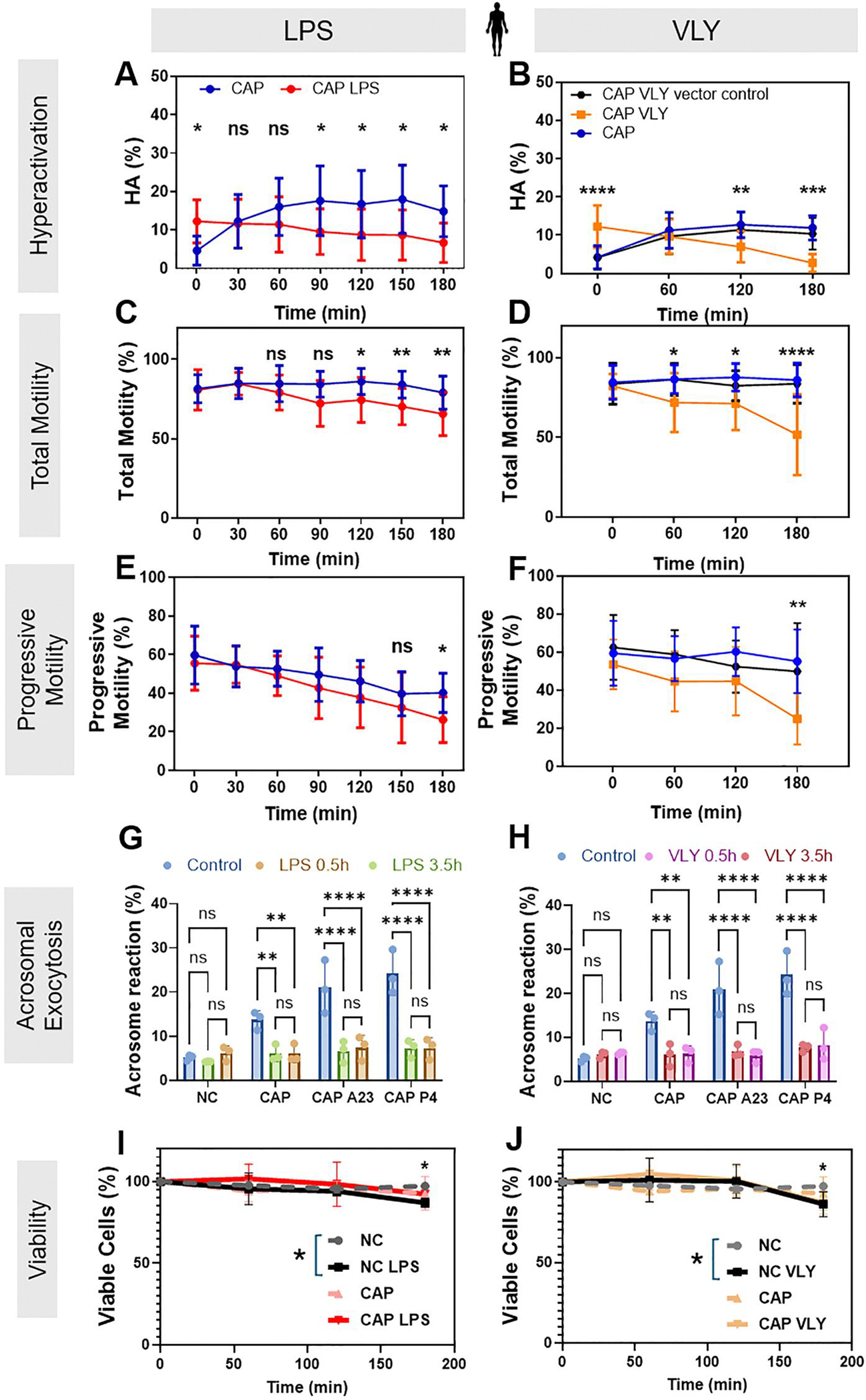
Lipopolysaccharide (LPS) and vaginolysin (VLY) impair human sperm hyperactivation, total motility, progressive motility, and acrosomal exocytosis in capacitating (CAP) conditions. CASA measurements were obtained for (**A, B**) hyperactivated motility (HA), (**C, D**) total motility, and (**E, F**) progressive motility of human sperm incubated under CAP conditions in the presence and absence of 0.1 μg/ml (**A, C, E**) LPS (n = 12 biological replicates for all experiments) or (**B, D, F**) VLY or VLY vector control (n = 12 biological replicates for all experiments). Zero minute is the timepoint of bovine serum albumin (BSA)+sodium bicarbonate addition to initiate *in vitro* capacitation. Acrosomal exocytosis (AE) was quantified in non-capacitating (NC), and CAP sperm (200 each, n = 3 biological replicates for **G, H**) incubated with (**G**) LPS or (**H**) VLY for 0.5 and 3.5 h. Similarly, AE induced by 10 μM A23187 (A23) or progesterone (P4), was also quantified in CAP sperm. Sperm viability for NC and CAP sperm was analyzed in the presence of 0.1 μg/ml (**I**) LPS or (**J**) VLY (10000 sperm each, n ≥ 3 biological replicates). Data were analyzed by independent *t*-test with Holm–Sidak’s multiple comparison test or two-way ANOVA with Bonferroni’s multiple comparison test and are presented as mean and SD. *P < 0.05, **P < 0.01, ***P < 0.001, ****P < 0.0001, ns, non-significant.

**Figure 4.**
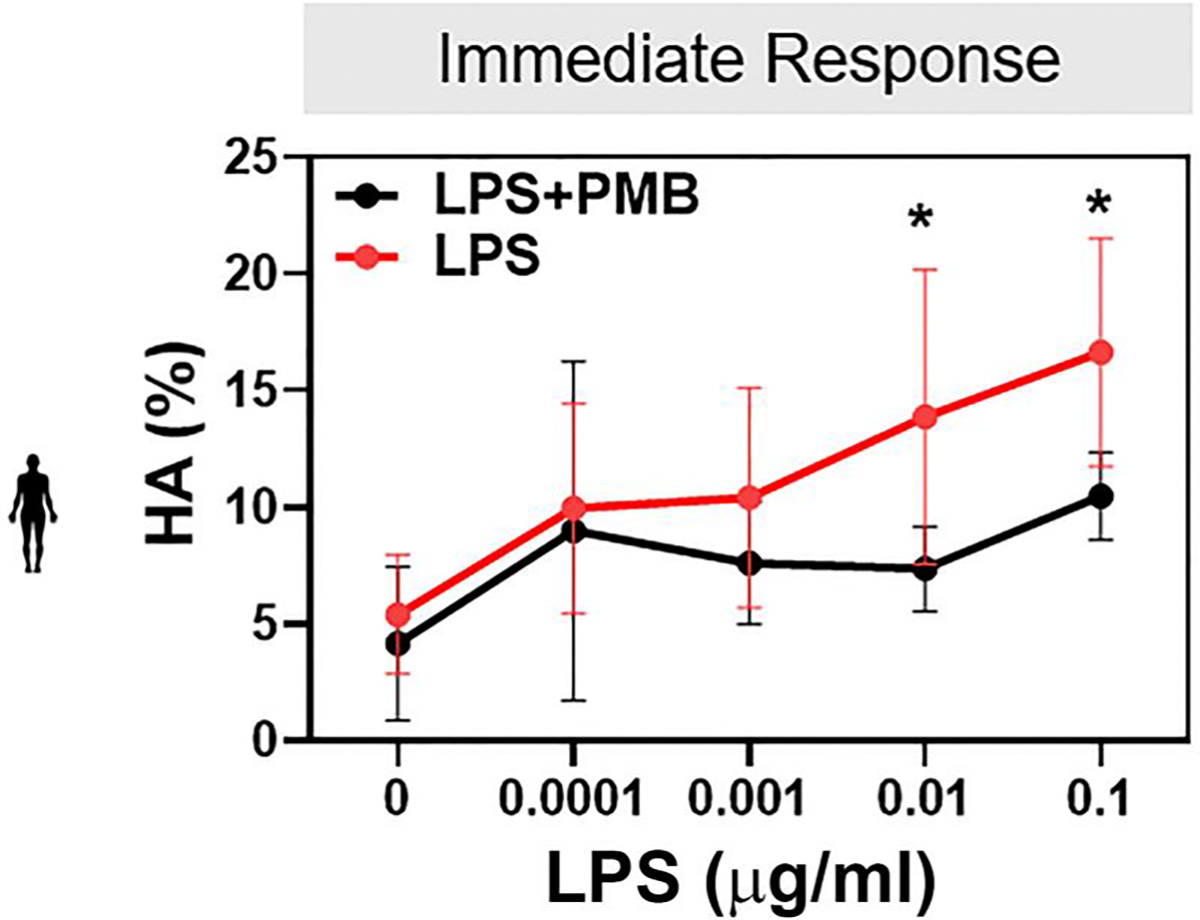
The initial lipopolysaccharide (LPS)-induced human sperm hyperactivation is dose-dependent and inhibited by polymyxin B (PMB). The percentage of sperm with hyperactivated motility (HA) at time 0 of capacitation measured by CASA in sperm samples incubated in the presence of increasing concentrations of LPS with or without 100 μg/ml PMB. Data are presented as mean and SD (n = 5 biological replicates). *P < 0.05 by unpaired *t*-test.

To determine whether LPS and VLY also impair human sperm function, we obtained leftover sperm samples from patients with normal semen parameters being seen at the Washington University in Saint Louis Fertility & Reproductive Medicine Center. As the median concentration of LPS in patients with BV is ~3000 EU/ml (Aroutcheva et al., 2008), we used 1000 EU/ml LPS, corresponding to 0.1 μg/ml LPS, for human sperm. The physiologically relevant concentration of VLY is unknown, so we used the same concentration as LPS. Within 2–5min (labeled as the ‘0 timepoint’ in Fig. 3A and B) after adding 0.1 μg/ml LPS or 0.1 μg/ml VLY to sperm in CAP conditions, the percentage of hyperactivated sperm was significantly greater than in untreated controls (Fig. 3A and B). However, by the end of the 180-min treatment period, human sperm treated with LPS or VLY showed significantly lower hyperactivated, total motility, and progressive motility than untreated sperm (Fig. 3A–F). LPS and VLY had no effect on human sperm hyperactivation or total or progressive motility in non-CAP conditions (Supplementary Fig. S5). PMB prevented the dose-dependent initial increase in human sperm hyperactivation induced by LPS (Fig. 4).

Next, we measured the effect of a 10-fold lower dose of LPS and VLY (0.1 μg/ml) on human sperm acrosomal exocytosis induced by addition of the Ca^2+^ ionophore A23187 or progesterone. As reported earlier (Baro Graf et al., 2020), about 15–30% of human sperm underwent induced acrosomal exocytosis in control conditions (Fig. 3G and H). Both LPS (Fig. 3G) and VLY (Fig. 3H) significantly reduced the percentage of acrosomal exocytosis induced by A23187 or progesterone in CAP conditions but had no effect in non-CAP conditions. At 180 min of treatment, LPS (Fig. 3I) and VLY (Fig. 3J) significantly reduced human sperm viability by 10% and 11.1%, respectively, relative to the controls in non-CAP conditions. In CAP conditions, LPS and VLY reduced the viability by 0.56% and 5.76%, respectively, but these changes were not statistically significant. We conclude that LPS and VLY impair human sperm function in a similar manner as they impair mouse sperm function.

### LPS and VLY rapidly and irreversibly induce increases in intracellular Ca^2+^ concentration in CAP mouse and human sperm

**Figure 5.**
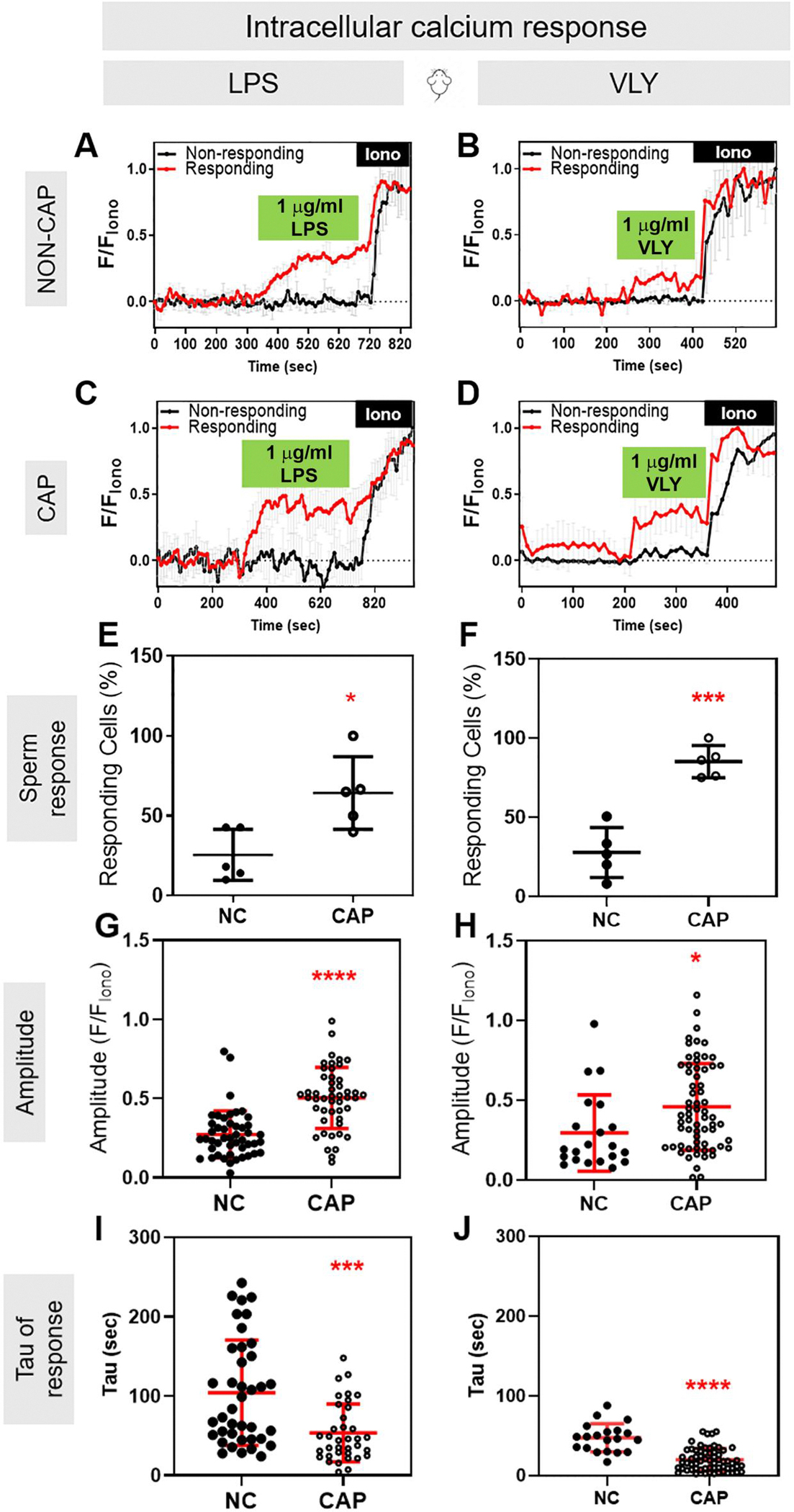
Lipopolysaccharide (LPS) and vaginolysin (VLY) cause rapid increases in intracellular calcium ([Ca^2+^]_i_) in capacitating (CAP) mouse sperm. Representative traces of normalized Fluo4-AM fluorescence in responding (red) versus non-responding (black) mouse sperm with 1 μg/ml (**A, C**) LPS or (**B, D**) VLY, incubated under (**A, B**) Non-capacitating (NC) or (**C, D**) CAP conditions. Each trace was normalized to its respective ionomycin (Iono, 5 μM) response. Percentages of mouse sperm responding to (**E**) LPS or (**F**) VLY, in NC or CAP conditions were calculated. (**G, H**) Amplitude and (**I, J**) tau of the [Ca^2+^]_i_ response in NC or CAP sperm, with 1 μg/ml (**G, I**) LPS and (**H, J**) VLY. Data are presented as mean and SD (n = 4 biological replicates for all experiments). *P < 0.05, ***P < 0.001, ****P < 0.0001, by unpaired *t*-test for (**E–J**).

**Figure 6.**
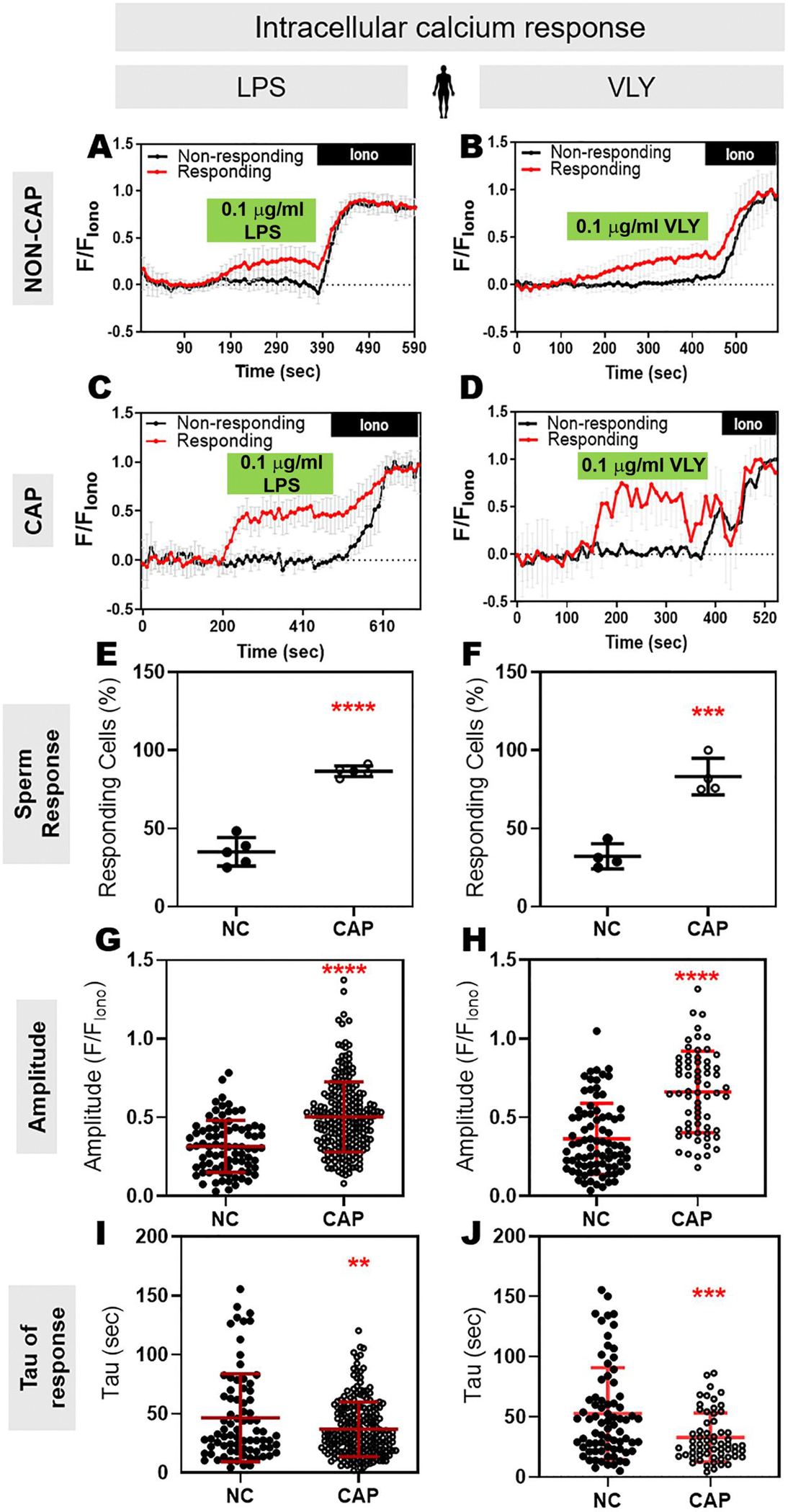
Lipopolysaccharide (LPS) and vaginolysin (VLY) cause rapid increases in intracellular calcium ([Ca^2+^]_i_) in capacitating (CAP) human sperm. Representative traces of normalized Fluo4-AM fluorescence in responding (red) versus non-responding (black) human sperm with 0.1 μg/ml (**A, C**) LPS or (**B, D**) VLY, incubated under (**A, B**) Non-capacitating (NC) or (**C, D**) CAP conditions. Each trace was normalized to its respective ionomycin (Iono, 5 μM) response. Percentages of human sperm responding to (**E**) LPS or (**F**) VLY, in NC or CAP conditions were calculated. (**G, H**) Amplitude and (**I, J**) tau of the [Ca^2+^]_i_ response in NC or CAP sperm, with 0.1 μg/ml (**G, I**) LPS or (**H, J**) VLY was quantified. Data are presented as mean and SD (n = 5 biological replicates for LPS, n = 4 biological replicates for VLY; in all experiments). *P < 0.01, ***P < 0.001, ****P < 0.0001, by unpaired *t*-test for (**E–J**).

**Figure 7.**
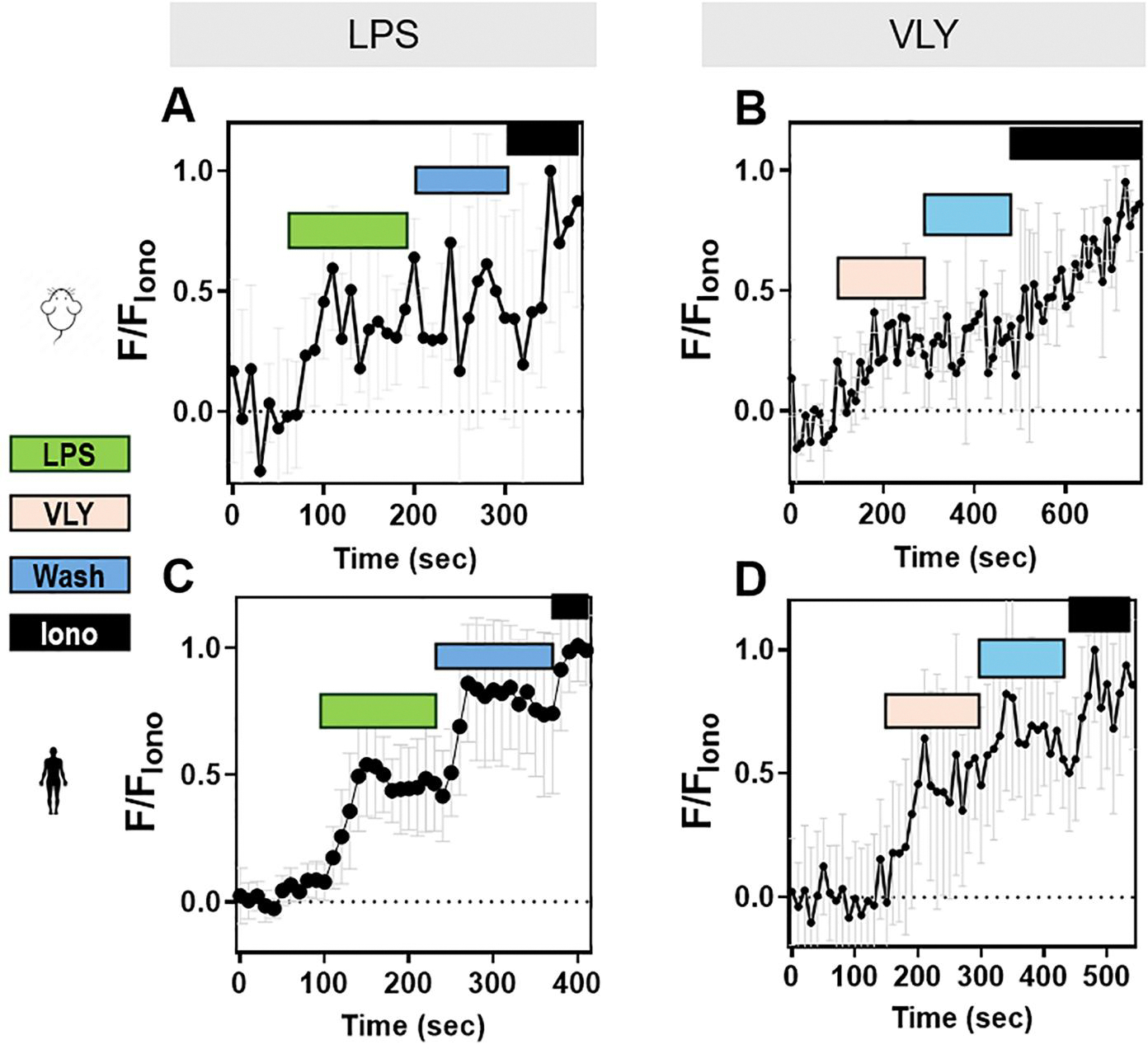
The lipopolysaccharide (LPS)- and vaginolysin (VLY)-induced intracellular calcium ([Ca^2+^]_i_) increases in mouse and human sperm are irreversible. Representative traces of normalized Fluo4-AM fluorescence in CAP (**A, B**) mouse or (**C, D**) human sperm, perfused with (**A, C**) LPS (denoted in green), or (**B, D**) VLY (denoted in pink), followed by media (Wash, denoted in blue), and subsequent perfusion with 5 μM ionomycin (Iono, denoted in black). Each trace was normalized to its respective ionomycin response. Data are presented as mean and SD (n = 3 biological replicates for all experiments).

Because both hyperactivated motility and acrosomal exocytosis depend on Ca^2+^ influx and increased intracellular Ca^2+^ concentration ([Ca^2+^]_i_), we wondered whether LPS and VLY affected the sperm [Ca^2+^]_i_. To test this idea, we loaded mouse sperm with the cell-permeant Ca^2+^-sensitive fluorescent dye Fluo-4 AM. In control conditions, the mouse sperm [Ca^2+^]_i_ reached a maximum after addition of CAP media, with a time constant (tau) of 164.2 ± 61.91. Addition of LPS and VLY increased [Ca^2+^]_i_ more rapidly, with a tau of 53.68 ± 36.36 and 20.01 ± 14.13, respectively (Supplementary Figs S6 and S7). Also, the amplitude of the increase in [Ca^2+^]_i_ was significantly larger in the presence of LPS (Supplementary Fig. S7). We next compared the LPS and VLY effects between non-CAP and CAP conditions. Between 10% and 40% of mouse sperm in non-CAP conditions and 50–100% of sperm in CAP conditions showed an increase in [Ca^2+^]_i_ after addition of LPS or VLY (responsive cells) (Fig. 5A–F). The amplitude of the Ca^2+^ response was greater and the tau was shorter in CAP than in non-CAP conditions (Fig. 5G–J) (see [Ca^2+^]_i_ responses induced by trace amounts of LPS- and VLY in Supplementary Fig. S8). Experiments using human sperm produced similar results (Fig. 6, Supplementary Fig. S9). Washout experiments indicated that the LPS- and VLY-induced Ca^2+^ responses were irreversible for both mouse and human sperm (Fig. 7). Therefore, we conclude that both LPS and VLY induced rapid and irreversible increases in [Ca^2+^]_i_, and these increases were significantly larger in capacitated conditions than in NC conditions.

### LPS-induced Ca^2+^ response requires TLR4 and external Ca^2+^ but not CatSper

**Figure 8.**
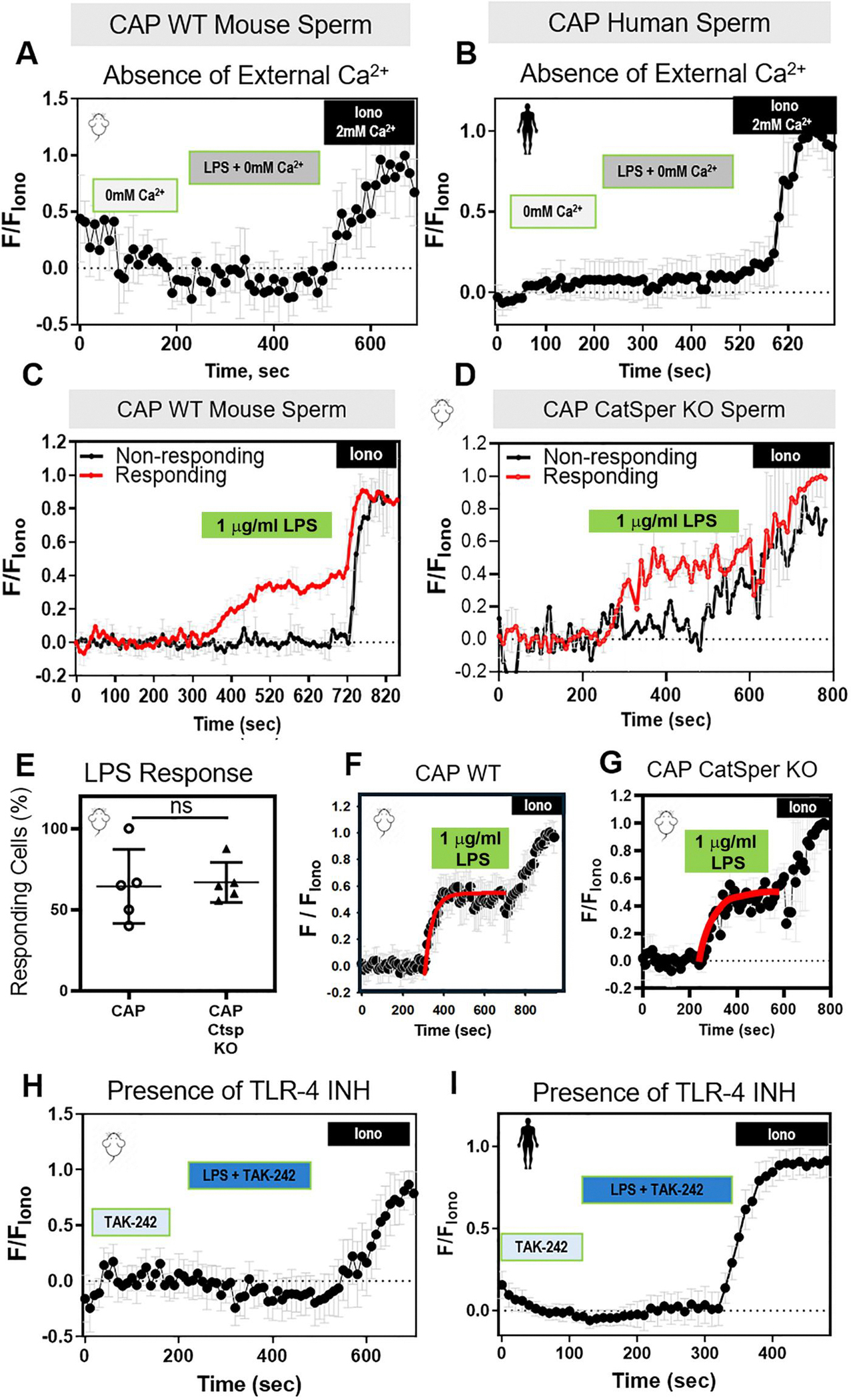
The lipopolysaccharide (LPS)-induced increase in sperm intracellular calcium ([Ca^2+^]_i_) is dependent on external Ca^2+^, independent of the CatSper ion channel, and mediated by the toll-like receptor 4 (TLR4). Representative traces of normalized Fluo4-AM fluorescence from capacitating (CAP) (**A**) wild-type (WT) mouse or (**B)** human sperm perfused with 0mM Ca^2+^, 0mM Ca^2+^ with LPS and ionomycin (Iono) with 2mM Ca^2+^ (n = 3 biological replicates). Representative traces for LPS responding (red) and non-responding (black) (**C**) WT or (**D**) CatSper knockout (KO) mouse sperm in the presence of 2mM extracellular Ca^2+^. (**E**) Percentages of LPS-responding sperm with increased [Ca^2+^]_i_ from WT and CatSper KO mice (n = 5 biological replicates). The LPS-induced [Ca^2+^]_i_ responses are shown on the traces obtained from (**F**) WT or (**G**) CatSper KO mouse sperm. Red curves are calculated as a standard exponential fit. Representative traces of Fluo4-AM fluorescence from CAP (**H**) WT mouse or (**I**) human sperm, perfused with 10 μM TAK-242 (TLR4 inhibitor), TAK-242 with LPS, and ionomycin (Iono), in the presence of 2 mM extracellular Ca^2+^ throughout the experiment (n = 3 biological replicates). Each trace was normalized to its respective ionomycin (Iono, 5 μM) response. Data are presented as mean and SD. ns, non-significant, by unpaired *t*-test in (**E**).

The main mechanism of [Ca^2+^]_i_ increase during sperm capacitation is Ca^2+^ influx through the sperm-specific Ca^2+^ channel, CatSper. To determine whether the effects of LPS required external Ca^2+^, we exposed mouse sperm to CAP conditions in media that lacked Ca^2+^. In this condition, LPS did not cause a [Ca^2+^]_i_ increase (Fig. 8A). Similarly, LPS did not increase [Ca^2+^]_i_ in human sperm in the absence of external Ca^2+^ (Fig. 8B). To determine whether CatSper was required for the LPS-induced [Ca^2+^]_i_ increase, we measured [Ca^2+^]_i_ in sperm from wild-type (Fig. 8C) and CatSper knockout (Fig. 8D) mice. Neither the percentages of sperm responding to LPS (Fig. 8E) nor the amplitude (Supplementary Fig. S10A) or tau (Supplementary Fig. S10B) of their responses differed significantly between sperm from wild-type and CatSper knockout mice. Moreover, the [Ca^2+^]_i_ changes in LPS-responding cells were similar for wild-type (Fig. 8F) and CatSper knockout (Fig. 8G) mouse sperm. These data indicate that LPS triggered an increase in [Ca^2+^]_i_ through a mechanism independent of CatSper.

Given that one of the mechanisms described for LPS-induced [Ca^2+^]_i_ increases in other cell types involves LPS binding to the TLR4 (Tauseef et al., 2012), we wondered whether TLR4 was required for LPS effects on [Ca^2+^]_i_ in sperm. To test this idea, we exposed sperm to LPS in the presence of the specific TLR4 inhibitor TAK-242 (Takashima et al., 2009; Yuko et al., 2020). The LPS-induced Ca^2+^ response did not occur in mouse (Fig. 8H) or human (Fig. 8I) sperm in the presence of 10 μM TAK-242. These data show that the [Ca^2+^]_i_ increase induced by LPS in human and mouse sperm is mediated by the TLR4 receptor. Furthermore, mouse sperm incubated with LPS and 10 μM TAK-242 showed no change in the hyperactivated motility (Supplementary Fig. S11), confirming the role of TLR4 in mediating the [Ca^2+^]_i_ increase and its downstream effect on sperm hyperactivation.

## Discussion

BV is more prevalent in infertile women than in fertile women, and 37.4% of females with unexplained infertility are diagnosed with BV (Salah et al., 2013). Although the cause of infertility among patients with BV is unclear, several mechanisms have been proposed. One possibility is that BV-related bacteria can induce immune activation and increase concentrations of proinflammatory cytokines, resulting in mucosal inflammation in the genital tract (van Teijlingen et al., 2020). Higher cervical concentrations of the cytokines interleukin (IL)-1β, IL-6, and IL-8 have been observed in women with infertility and BV (Spandorfer et al., 2001). Another possibility is that sialidases and other mucinases produced by microorganisms impair cervical mucus integrity (Wiggins et al., 2001) by degrading mucins. This could weaken the natural barrier against microbial invasion and facilitate bacterial adhesion and colonization, possibly leading to upper reproductive tract disease and infertility. Finally, women with BV have a 3.4- and 4.1-fold, respectively, elevated risk of acquiring the sexually transmitted infections with *Chlamydia trachomatis* and *Neisseria gonorrhoeae* (Wiesenfeld et al., 2003), which can impair fertility. Additionally, BV is associated with increased rates of upper genital tract infections and pelvic inflammatory disease, both of which are linked to infertility (Ravel et al., 2021).

Here, we present three lines of evidence supporting a new mechanism by which BV contributes to female infertility, i.e. the BV-associated toxins LPS and VLY interfere with sperm capacitation, which is required to fertilize an oocyte. First, both LPS and VLY treatments reduced the fertilizing ability of mouse sperm. Second, LPS and VLY impaired hyperactivation, motility, and acrosomal exocytosis in both mouse and human sperm. Third, LPS and VLY caused premature and irreversible increases in intracellular Ca^2+^ concentration ([Ca^2+^]_i_) in both mouse and human sperm, that could be damaging to sperm function.

Our results regarding the impact of LPS on mouse sperm are consistent with findings reported by Fujita et al. (2011). They reported decreased mouse sperm motility, increased sperm apoptosis, and decreased fertilization ability after 6 h of incubation with LPS. They also showed that PMB prevented the negative effects of LPS. We observed that incubation with LPS for over 90 min led to decreased sperm motility and a nearly 50% reduction in the formation of two-celled embryos. This effect was mitigated by PMB. We also noted a slight decrease in viability after 3 h in capacitated media, with no statistical significance. However, the largest effects we observed were on hyperactivated motility and acrosomal exocytosis, which were not explored by Fujita and co-workers.

Li et al. (2016) also found that LPS decreased human sperm motility and intracellular cAMP concentrations. Inconsistent with our findings, Li et al. (2016) reported that LPS did not affect sperm [Ca^2+^]_i_, acrosomal exocytosis, or capacitation. One possible explanation for these negative results could be a contamination of controls or reagents with LPS, which is commonly seen in commercial-grade reagents (Weinstein et al., 2008). Alternatively, LPS can adhere to tubes upon dilution and be lost. We used methods to prevent these problems and found that the effects of LPS were irreversible. Given this irreversibility, LPS in the vagina in patients with BV could affect sperm fertility. Consistent with our findings, Sahnoun et al. reported that LPS led to reduced percentages of sperm acrosomal exocytosis in infertile males (Sahnoun et al., 2017). Additionally, LPS was reported to decrease human sperm motility and curvilinear movement, inhibit phosphorylation of NF-κB inhibitor alpha, and cause increased β-oxidation of fatty acids (Li et al., 2023).

Previous results regarding the effects of LPS on human sperm viability have been contradictory. In one report, 0.1–100 μg/ml LPS had no effect on viability (Li et al., 2016), whereas in another report, 50 μg/ml LPS caused 80% reduction in human sperm viability (Galdiero et al., 1988). With our validated concentrations of high-quality LPS and precautions to avoid loss of LPS through adsorption, we observed only a small decrease in sperm viability after 180 min of treatment. This small effect is unlikely to account for the effects of LPS on motility or acrosomal exocytosis. Most previous studies used LPS from *E. coli*, as we also did here. The lipid A moiety of *Prevotella intermedia* LPS activates murine cells through a TLR4-dependent pathway, similar to *E. coli*-type lipid A (Hashimoto et al., 2003). Therefore, using LPS from *E. coli*, rather than from *Prevotella*, would likely yield similar findings.

Both sperm hyperactivation and acrosomal exocytosis are finely regulated by intracellular Ca^2+^. This Ca^2+^ entry is required for both the initiation and maintenance of hyperactivation through direct regulation of axonemal machinery components (Carlson et al., 2003). However, excessive increases in [Ca^2+^]_i_ in mammalian sperm are associated with impaired fertilization potential. This includes mitochondrial failure, motility loss, activation of apoptotic cascades, and premature loss of the acrosome (Liu and Baker, 1990). Prematurely inducing the human acrosome reaction with the Ca^2+^ ionophore A23187 also decreases sperm-zona pellucida binding, and cause fertilization failure *in vitro* (Liu and Baker, 1990). Irreversible increases in [Ca^2+^]_i_ seen in spermatozoa deficient in the Ca^2+^ pump PMCA4, responsible for extruding Ca^2+^, results in male infertility (Okunade et al., 2004). Furthermore, high concentrations (10–20 μM) of the Ca^2+^ ionophore A23187 initially increase the sperm flagellar beat, then immobilize sperm after 10 min. Concentrations of 5–10 μM A23187 rapidly immobilized sperm by elevating [Ca^2+^]_i_, whereas lower concentrations (0.5 and 1 μM) caused smaller [Ca^2+^]_i_ increases and resulted in hyperactivation. Notably, A23187 in Ca^2+^-free medium did not immobilize sperm. These findings suggest that excessive Ca^2+^ influx affects motility and, at high concentrations, can lead to sperm immobilization. We found that hyperactivation in human sperm increased within 2–5min after sperm were suspended in capacitation media and exposed to LPS and VLY. This early hyperactivation was triggered by a rapid Ca^2+^ influx, which also occurred within 2–3 min after exposure to LPS and VLY. These fast, high-amplitude, and irreversible [Ca^2+^]_i_ increases in sperm exposed to LPS and VLY might impair sperm function. However, the mechanisms by which this irreversible [Ca^2+^]_i_ occurs in response to LPS and VLY might be distinct.

In mammalian sperm, the Ca^2+^ influx into the flagellum during capacitation is primarily regulated by CatSper (Kirichok et al., 2006), but we found that LPS-induced Ca^2+^ influx in mouse sperm was independent of CatSper. Fujita et al. reported that the negative effects of LPS on sperm motility and fertility were blocked in TLR4 knockout mice (Fujita et al., 2011), suggesting that TLR4 participates in LPS-mediated Ca^2+^ signaling. Our experiments with the TLR4 inhibitor support the idea that the LPS-induced [Ca^2+^]_i_ increase and the downstream effect on sperm hyperactivation was mediated by the TLR4 receptor. Since CatSper was not involved, we speculate that in response to LPS, Ca^2+^ enters either through store-operated Ca^2+^ channels or voltage-gated Ca^2+^ channels such as CaV3.2 or the transient receptor potential vanilloid 4 (TRPV4) channel (Darszon et al., 2011; Brown et al., 2019). We are currently investigating which of these could be responsible for the LPS-induced intracellular Ca^2+^ increase.

VLY is a cytolysin that forms pores in the cell membrane (Ragaliauskas et al., 2019). In addition to causing cells to leak their intracellular contents (Randis et al., 2013; Morrill et al., 2023), the disruptions to the membrane would likely allow extracellular Ca^2+^ to enter the cytoplasm, as we observed in both mouse and human sperm. Because VLY embeds in the human cell membrane, it is expected to be irreversible. Thus, it was not surprising that we were unable to wash out the effects of VLY in sperm. Given the acute and long-term effects on sperm function seen with LPS and VLY, we anticipate that both toxins likely cause sperm ‘energy burnout’ by triggering excessive Ca^2+^ influx, disrupting mitochondrial function, and depleting ATP (Zhu et al., 2016). Future experiments will be conducted to explore these mechanisms.

In conclusion, our *in vitro* results show that the BV-associated toxins LPS and VLY modulate sperm Ca^2+^ entry, leading to dysregulated hyperactivation, inhibition of acrosomal exocytosis, and impaired fertility. If these effects occur *in vivo*, then BV toxins in the vagina could impair key events necessary to prepare sperm for fertilization. This mechanism may contribute to infertility in patients with BV.

## Supplementary Material

Supp Table

Supp Fig S1

Supp Fig S2

Supp Fig S3

Supp Fig S4

Supp Fig S5

Supp Fig S6

Supp Fig S7

Supp Fig S8

Supp Fig S9

Supp Fig S10

Supp Fig S11

Supplementary data

Supplementary data are available at *Human Reproduction* online.

## Data Availability

All data are available in the main text or the supplementary materials.

## References

[R1] Allsworth JE, Peipert JF. Prevalence of Bacterial Vaginosis: 2001–2004 National Health and Nutrition Examination Survey data. Obstet Gynecol 2007;109:114–120. 17197596 10.1097/01.AOG.0000247627.84791.91

[R2] Amsel R, Totten PA, Spiegel CA, Chen KCS, Eschenbach D, Holmes KK. Nonspecific Vaginitis Diagnostic Criteria and Microbial and Epidemiologic Associations. Am J Med 1983;74:14–22. 6600371 10.1016/0002-9343(83)91112-9

[R3] Aroutcheva A, Zaodung L, Faro S. Prevotella bivia as a Source of Lipopolysaccharide in the Vagina. Anaerobe 2008;14:256–260. 18849004 10.1016/j.anaerobe.2008.08.002 PMC2651005

[R4] Baro Graf C, Ritagliati C, Torres-Monserrat V, Stival C, Carizza C, Buffone MG, Krapf D. Membrane potential assessment by fluorimetry as a predictor tool of human sperm fertilizing capacity. Front Cell Dev Biol 2020;7:383. 32010695 10.3389/fcell.2019.00383 PMC6979052

[R5] Brown SG, Publicover SJ, Barratt CLR, Martins da Silva SJ. Human sperm ion channel (dys)function: implications for fertilization. Hum Reprod Update 2019;25:758–776. 31665287 10.1093/humupd/dmz032 PMC6847974

[R6] Campisciano G, Iebba V, Zito G, Luppi S, Martinelli M, Fischer L, De Seta F, Basile G, Ricci G, Comar M. *Lactobacillus iners* and *gasseri*, *Prevotella bivia* and HPV belong to the microbiological signature negatively affecting human reproduction. Microorganisms 2021; 9: 39. 10.3390/microorganisms9010039 PMC7824525 33375526

[R7] Carlson AE, Westenbroek RE, Quill T, Ren D, Clapham DE, Hille B, Garbers DL, Babcock DF. CatSper1 required for evoked Ca2+ entry and control of flagellar function in sperm. Proc Natl Acad Sci USA 2003;100:14864–14868. 14657352 10.1073/pnas.2536658100 PMC299831

[R8] Chávez JC, Ferreira JJ, Butler A, VegaBeltrán L, De JL, Treviño CL, Darszon A, Salkoff L, Santi CM. SLO3 K+ channels control calcium entry through CATSPER channels in sperm. J Biol Chem 2014;289:32266–32275. 25271166 10.1074/jbc.M114.607556 PMC4231700

[R9] Cocomazzi G, De Stefani S, Del Pup L, Palini S, Buccheri M, Primiterra M, Sciannamè N, Faioli R, Maglione A, Baldini GM The Impact of the Female Genital Microbiota on the Outcome of Assisted Reproduction Treatments. Microorganisms 2023;11:1443. 37374945 10.3390/microorganisms11061443 PMC10302789

[R10] Darszon A, Nishigaki T, Beltran C, Treviño CL. Calcium channels in the development, maturation, and function of spermatozoa. Physiol Rev 2011;91:1305–1355. 22013213 10.1152/physrev.00028.2010

[R11] De La Vega-Beltran JL, Sánchez-Cárdenas C, Krapf D, Hernandez-González EO, Wertheimer E, Treviño CL, Visconti PE, Darszon A. Mouse sperm membrane potential hyperpolarization is necessary and sufficient to prepare sperm for the acrosome reaction. J Biol Chem 2012;287:44384–44393. 23095755 10.1074/jbc.M112.393488 PMC3531752

[R12] Eckert LO, Moore DE, Patton DL, Agnew KJ, Eschenbach DA. Relationship of vaginal bacteria and inflammation with conception and early pregnancy loss following in-vitro fertilization. Infect Dis Obs Gynecol 2003;11:11–17. 10.1155/S1064744903000024 PMC1852261 12839628

[R13] Ferreira JJ, Cassina A, Irigoyen P, Ford M, Pietroroia S, Peramsetty N, Radi R, Santi CM, Sapiro R. Increased mitochondrial activity upon CatSper channel activation is required for mouse sperm capacitation. Redox Biol 2021;48:102176. 34753004 10.1016/j.redox.2021.102176 PMC8585656

[R14] Firmal P, Shah VK, Chattopadhyay S. Insight Into TLR4-Mediated Immunomodulation in Normal Pregnancy and Related Disorders. Front Immunol 2020;11:807. 32508811 10.3389/fimmu.2020.00807 PMC7248557

[R15] Fujita Y, Mihara T, Okazaki T, Shitanaka M, Kushino R, Ikeda C, Negishi H, Liu Z, Richards JS, Shimada M. Toll-like receptors (TLR) 2 and 4 on human sperm recognize bacterial endotoxins and mediate apoptosis. Hum Reprod 2011;26:2799–2806. 21775336 10.1093/humrep/der234 PMC3174031

[R16] Galdiero F, Gorga F, Bentivoglio C, Mancuso R, Galdiero E, Tufano MA. The action of LPS porins and peptidoglycan fragments on human spermatozoa. Infection 1988;16:349–353. 2851555 10.1007/BF01644545

[R17] George SD, Van Gerwen OT, Dong C, Sousa LGV, Cerca N, Elnaggar JH, Taylor CM, Muzny CA. The Role of Prevotella Species in Female Genital Tract Infections. Pathogens 2024;13:364. 38787215 10.3390/pathogens13050364 PMC11123741

[R18] Haahr T, Zacho J, Brauner M, Shathmigha K, Skov Jensen J, Humaidan P. Reproductive outcome of patients undergoing in vitro fertilisation treatment and diagnosed with bacterial vaginosis or abnormal vaginal microbiota : a systematic PRISMA review and meta-analysis. BJOG 2019;126:200–207. 29469992 10.1111/1471-0528.15178

[R19] Hashimoto M, Asai Y, Tamai R, Jinno T, Umatani K, Ogawa T. Chemical structure and immunobiological activity of lipid A from Prevotella intermedia ATCC 25611 lipopolysaccharide. FEBS Lett 2003;543:98–102. 12753913 10.1016/s0014-5793(03)00414-9

[R20] Hasuwa H, Muro Y, Ikawa M, Kato N, Tsujimoto Y, Okabe M. Transgenic mouse sperm that have green acrosome and red mitochondria allow visualization of sperm and their acrosome reaction in vivo. Exp Anim 2010;59:105–107. 20224175 10.1538/expanim.59.105

[R21] Hong X, Ma J, Yin J, Fang S, Geng J, Zhao H, Zhu M, Ye M, Zhu X, Xuan Y The association between vaginal microbiota and female infertility : a systematic review and meta - analysis. Arch Gynecol Obstet 2020;302:569–578. 32638096 10.1007/s00404-020-05675-3

[R22] Kirichok Y, Navarro B, Clapham DE. Whole-cell patch-clamp measurements of spermatozoa reveal an alkaline-activated Ca2+ channel. Nature 2006;439:737–740. 16467839 10.1038/nature04417

[R23] Łaniewski P, Herbst-Kralovetz MM. Bacterial vaginosis and health-associated bacteria modulate the immunometabolic landscape in 3D model of human cervix. NPJ Biofilms Microbiomes 2021;7:1–17. 34903740 10.1038/s41522-021-00259-8 PMC8669023

[R24] Li Y, Hu Y, Wang Z, Lu T, Yang Y, Diao H, Zheng X, Xie C, Zhang P, Zhang X IKBA phosphorylation governs human sperm motility through ACC-mediated fatty acid beta-oxidation. Commun Biol 2023;6:1–12. 36966253 10.1038/s42003-023-04693-6 PMC10039860

[R25] Li Z, Zhang D, He Y, Ding Z, Mao F, Luo T, Zhang X. Lipopolysaccharide compromises human sperm function by reducing intracellular cAMP. Tohoku J Exp Med 2016;238:105–112. 26782775 10.1620/tjem.238.105

[R26] Liu DY, Baker HWG. Inducing the human acrosome reaction with a calcium ionophore A23187 decreases sperm-zona pellucida binding with oocytes that failed to fertilize in vitro. J Reprod Fertil 1990;89:127–134. 2115581 10.1530/jrf.0.0890127

[R27] Lyon M, Li P, Ferreira J, Lazarenko R, Kharade S, Kramer M, McClenahan S, Days E, Bauer J, Spitznagel B A selective inhibitor of the sperm-specific potassium channel SLO3 impairs human sperm function. Proc Natl Acad Sci USA 2023;120:e2212338120. 36649421 10.1073/pnas.2212338120 PMC9942793

[R28] Magata F, Tsukamura H, Matsuda F. The impact of inflammatory stress on hypothalamic kisspeptin neurons : mechanisms underlying inflammation-associated infertility in humans and domestic animals. Peptides 2023;162:170958. 36682622 10.1016/j.peptides.2023.170958

[R29] Mania-Pramanik J, Kerkar ÃSC, Mehta PB, Potdar S, Salvi VS. Use of Vaginal pH in Diagnosis of Infections and Its Association With Reproductive Manifestations. J Clin Lab Anal 2008;22:375–379. 18803273 10.1002/jcla.20273 PMC6649105

[R30] Molina LCP, Gunderson S, Riley J, Lybaert P, Borrego-Alvarez A, Jungheim ES, Santi CM. Membrane potential determined by flow cytometry predicts fertilizing ability of human sperm. Front Cell Dev Biol 2020;7:387. 32039203 10.3389/fcell.2019.00387 PMC6985285

[R31] Molina LCP, Luque GM, Balestrini PA, Marín-Briggiler CI, Romarowski A, Buffone MG. Molecular basis of human sperm capacitation. Front Cell Dev Biol 2018;6:72. 30105226 10.3389/fcell.2018.00072 PMC6078053

[R32] Morrill SR, Saha S, Varki AP, Lewis WG, Ram S, Lewis AL. Gardnerella vaginolysin potentiates glycan molecular mimicry by Neisseria gonorrhoeae. J Infect Dis 2023;228:1610–1620. 37722688 10.1093/infdis/jiad391 PMC10681867

[R33] Mortimer ST, Swan MA, Mortimer D. Effect of seminal plasma on capacitation and hyperactivation in human spermatozoa. Hum Reprod 1998;13:2139–2146. 9756285 10.1093/humrep/13.8.2139

[R34] Murphy K, Mitchell CM. The Interplay of Host Immunity, Environment and the Risk of Bacterial Vaginosis and Associated Reproductive Health Outcomes. J Infect Dis 2016;214:S29–S35. 27056955 10.1093/infdis/jiw140 PMC4957509

[R35] Nakagata N Cryopreservation of mouse spermatozoa and in vitro fertilization. Methods Mol Biol 2011:693:57–73. 21080274 10.1007/978-1-60761-974-1_4

[R36] O’Doherty AM, Di Fenza M, Kölle S. Lipopolysaccharide (LPS) disrupts particle transport, cilia function and sperm motility in an ex vivo oviduct model. Sci Rep 2016;6:24583. 27079521 10.1038/srep24583 PMC4832340

[R37] Okunade GW, Miller ML, Pyne GJ, Sutliff RL, O’Connor KT, Neumann JC, Andringa A, Miller DA, Prasad V, Doetschman T Targeted ablation of plasma membrane Ca2+-ATPase (PMCA) 1 and 4 indicates a major housekeeping function for PMCA1 and a critical role in hyperactivated sperm motility and male fertility for PMCA4. J Biol Chem 2004;279:33742–33750. 15178683 10.1074/jbc.M404628200

[R38] Ragaliauskas T, Plečkaitytė M, Jankunec M, Labanauskas L, Baranauskiene L, Valincius G. Inerolysin and vaginolysin, the cytolysins implicated in vaginal dysbiosis, differently impair molecular integrity of phospholipid membranes. Sci Rep 2019; 9:10606. 31337831 10.1038/s41598-019-47043-5 PMC6650466

[R39] Randis TM, Kulkarni R, Aguilar JL, Ratner AJ. Antibody-based detection and inhibition of vaginolysin, the Gardnerella vaginalis cytolysin. PLoS One 2009;4:e5207. 19370149 10.1371/journal.pone.0005207 PMC2666159

[R40] Randis TM, Zaklama J, LaRocca TJ, Los FCO, Lewis EL, Desai P, Rampersaud R, Amaral FE, Ratner AJ. Vaginolysin drives epithelial ultrastructural responses to *Gardnerella vaginalis*. Infect Immun 2013;81:4544–4550. 24082080 10.1128/IAI.00627-13 PMC3837968

[R41] Ravel J, Gajer P, Abdo Z, Schneider GM, Koenig SSK, McCulle SL, Karlebach S, Gorle R, Russell J, Tacket CO Vaginal microbiome of reproductive-age women. Proc Natl Acad Sci USA 2011;108:4680–4687. 20534435 10.1073/pnas.1002611107 PMC3063603

[R42] Ravel J, Moreno I, Simón C. Bacterial vaginosis and its association with infertility, endometritis, and pelvic inflammatory disease. Am J Obstet Gynecol 2021;224:251–257. 33091407 10.1016/j.ajog.2020.10.019

[R43] Sahnoun S, Sellami A, Chakroun N, Mseddi M, Attia H, Rebai T, Lassoued S. Human sperm Toll-like receptor 4 (TLR4) mediates acrosome reaction, oxidative stress markers, and sperm parameters in response to bacterial lipopolysaccharide in infertile men. J Assist Reprod Genet 2017;34:1067–1077. 28550386 10.1007/s10815-017-0957-8 PMC5533687

[R44] Salah RM, Allam AM, Magdy AM, Mohamed AS. Bacterial vaginosis and infertility: cause or association? Eur J Obstet Gynecol Reprod Biol 2013;167:59–63. 23199811 10.1016/j.ejogrb.2012.10.031

[R45] Salminen A, Paananen R, Vuolteenaho R, Metsola J, Ojaniemi M, Autio-Harmainen H, Hallman M. Maternal endotoxin-induced preterm birth in mice: fetal responses in toll-like receptors, collectins, and cytokines. Pediatr Res 2008;63:280–286. 18287966 10.1203/PDR.0b013e318163a8b2

[R46] Sosa CM, Pavarotti MA, Zanetti MN, Zoppino FCM, De Blas GA, Mayorga LS. Kinetics of human sperm acrosomal exocytosis. Mol Hum Reprod 2015;21:244–254. 25452326 10.1093/molehr/gau110

[R47] Spandorfer SD, Neuer A, Giraldo PC, Rosenwaks Z, Witkin SS. Relationship of abnormal vaginal flora, proinflammatory cytokines and idiopathic infertility in women undergoing IVF. J Reprod Med 2001;46:806–810. 11584481

[R48] Suarez SS. Control of hyperactivation in sperm. Hum Reprod Update 2008;14:647–657. 18653675 10.1093/humupd/dmn029

[R49] Takashima K, Matsunaga N, Yoshimatsu M, Hazeki K, Kaisho T, Uekata M, Hazeki O, Akira S, Iizawa Y, Ii M. Analysis of binding site for the novel small-molecule TLR4 signal transduction inhibitor TAK-242 and its therapeutic effect on mouse sepsis model Abbreviations. Br J Pharmacol 2009;157:1250–1262. 19563534 10.1111/j.1476-5381.2009.00297.x PMC2743844

[R50] Tauseef M, Knezevic N, Chava KR, Smith M, Sukriti S, Gianaris N, Obukhov AG, Vogel SM, Schraufnage DE, Dietrich A TLR4 activation of TRPC6-dependent calcium signaling mediates endotoxininduced lung vascular permeability and inflammation. J Exp Med 2012;209:1953–1968. 23045603 10.1084/jem.20111355 PMC3478927

[R51] Turpin R, Tuddenham S, He X, Klebanoff MA, Ghanem KG, Brotman RM. Bacterial Vaginosis and Behavioral Factors Associated with Incident Pelvic Inflammatory Disease in the Longitudinal Study of Vaginal Flora. J Infect Dis 2021;224:S137–S144. 34396403 10.1093/infdis/jiab103 PMC8499701

[R52] van Oostrum N, De Sutter P, Meys J, Verstraelen H. Risks associated with bacterial vaginosis in infertility patients : a systematic review and meta-analysis. Hum Reprod 2013;28:1809–1815. 23543384 10.1093/humrep/det096

[R53] van Teijlingen NH, Helgers LC, Zijlstra-Willems EM, van Hamme JL, Ribeiro CMS, Strijbis K, Geijtenbeek TBH. Vaginal dysbiosis associated-bacteria *Megasphaera elsdenii* and *Prevotella timonensis* induce immune activation via dendritic cells. J Reprod Immunol 2020;138:103085. 32004804 10.1016/j.jri.2020.103085

[R54] Wang X, Rousset CI, Hagberg H, Mallard C. Lipopolysaccharide-induced inflammation and perinatal brain injury. Semin Fetal Neonatal Med 2006;11:343–353. 16793357 10.1016/j.siny.2006.04.002

[R55] Weinstein JR, Swarts S, Bishop C, Hanisch K, Möller T. Lipopolysaccharide is a Frequent and Significant Contaminant in Microglia-Activating Factors. Glia 2008;56:16–26. 17910052 10.1002/glia.20585 PMC2926344

[R56] Wiesenfeld HC, Hillier SL, Krohn MA, Landers DV, Sweet RL. Bacterial Vaginosis Is a Strong Predictor of Neisseria gonorrhoeae and Chlamydia trachomatis Infection. Clin Infect Dis 2003;36:663–668. 12594649 10.1086/367658

[R57] Wiggins R, Hicks SJ, Soothill PW, Millar MR, Corfield AP. Mucinases and sialidases: their role in the pathogenesis of sexually transmitted infections in the female genital tract. Sex Transm Infect 2001;77:402–408. 11714935 10.1136/sti.77.6.402 PMC1744407

[R58] Yanagimachi R Mammalian fertilization. In: Knobil E, Neill JD (eds). The Physiology of Reproduction New York: Raven Press, 1994, 189–317.

[R59] Yoo DK, Lee S-H. Effect of lipopolysaccharide (LPS) exposure on the reproductive organs of immature female rats. Dev Reprod 2016;20:113–121. 27660826 10.12717/DR.2016.20.2.113 PMC5027216

[R60] Yuko O, Yuko M, Masafumi S, Kazuho S, Shoichiro H, Shimomura K, Inoue S, Kotani J. TAK-242, a specific inhibitor of Toll-like receptor 4 signalling, prevents endotoxemia-induced skeletal muscle wasting in mice. Sci Rep 2020;10:694. 31959927 10.1038/s41598-020-57714-3 PMC6970997

[R61] Zhu X, Shi D, Li X, Gong W, Wu F, Guo X, Xiao H, Liu L, Zhou H. TLR signalling affects sperm mitochondrial function and motility via phosphatidylinositol 3-kinase and glycogen synthase kinase-3α. Cell Signal 2016;28:148–156. 26658093 10.1016/j.cellsig.2015.12.002

