# Supplementary material for: Bacterial vaginosis toxins impair sperm capacitation and fertilization
Source: Hum Reprod. 2025 Jul 13;40(9):1720–34. doi: 10.1093/humrep/deaf132 (PMC12370371; doi:10.1093/humrep/deaf132)
Supplement: deaf132_Supplementary_Figure_S1 [file deaf132_supplementary_figure_s1.pdf]

Human Reproduction, 2025, 1–1
https://doi.org/10.1093/humrep/deaf132
Supplementary Data

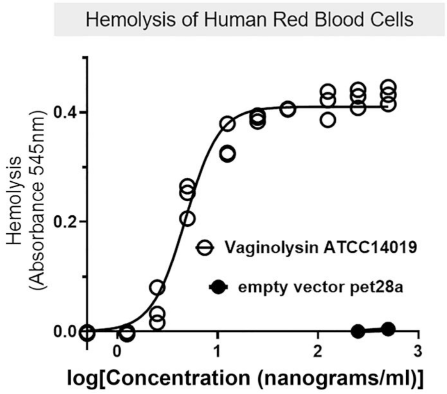

Supplementary Figure S1. Hemolysis activity of vaginolysin (VLY) from *Gardnerella vaginalis* ATCC14019 and the empty pet28a vector against human red blood cells. Human red blood cells were incubated in the presence of the purified VLY in concentrations of (0.50–500 ng/ml) and the empty pet28a vector mock purification. After spinning out the intact cells, hemolysis activity was measured at an absorbance of 545 nm with three replicates.
