# Supplementary material for: Bacterial vaginosis toxins impair sperm capacitation and fertilization
Source: Hum Reprod. 2025 Jul 13;40(9):1720–34. doi: 10.1093/humrep/deaf132 (PMC12370371; doi:10.1093/humrep/deaf132)
Supplement: deaf132_Supplementary_Figure_S2 [file deaf132_supplementary_figure_s2.pdf]

OXFORD
human reproduction
Human Reproduction, 2025, 1–1
https://doi.org/10.1093/humrep/deaf132
Supplementary Data

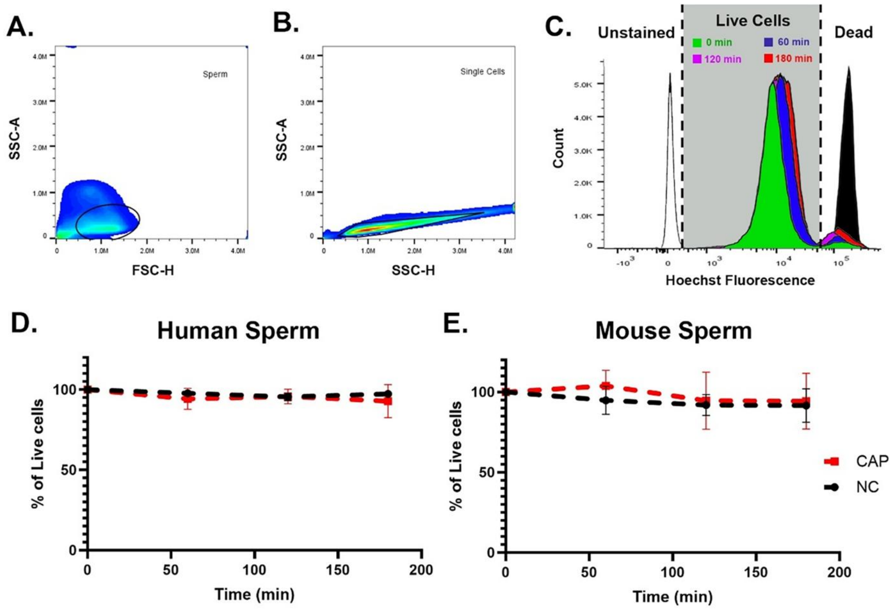

Supplementary Figure S2. Viability assay work-flow for human and mouse sperm. (**A**) Cell size (forward scatter height, FSC-H) and granularity (side scatter area, SSC-A) were used to identify the main cell population. (**B**) SSC-A versus side scatter height (SSC-H) was used to distinguish the single-cell population. (**C**) Histogram of Hoechst fluorescence showing unstained cells, bleach-treated dead cells (3% bleach), and cells incubated for different durations (0, 60, 120, and 180 min). (**D, E**) Viability of sperm under non-capacitating (NC) and capacitating (CAP) conditions over time (0, 60, 120, and 180 min) for human (**D**) and mouse (**E**) sperm. Data were normalized to T0 for each condition.
