# Supplementary material for: Bacterial vaginosis toxins impair sperm capacitation and fertilization
Source: Hum Reprod. 2025 Jul 13;40(9):1720–34. doi: 10.1093/humrep/deaf132 (PMC12370371; doi:10.1093/humrep/deaf132)
Supplement: deaf132_Supplementary_Figure_S3 [file deaf132_supplementary_figure_s3.pdf]

OXFORD
human reproduction

Human Reproduction, 2025, 1–1
https://doi.org/10.1093/humrep/deaf132
Supplementary Data

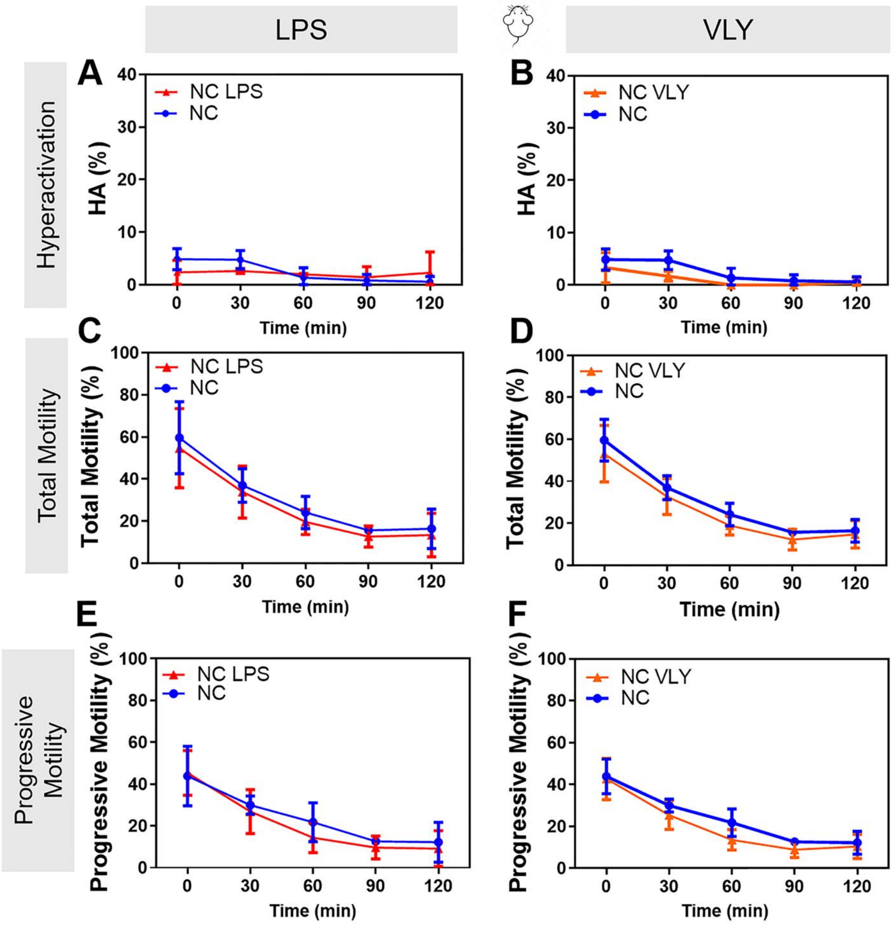

Supplementary Figure S3. Lipopolysaccharide (LPS) and vaginolysin (VLY) do not affect mouse sperm hyperactivated, total motility, or progressive motility in non-capacitating (NC) conditions. CASA measurements of (**A, B**) hyperactivated (HA), (**C, D**) total motility, and (**E, F**) progressive motility of mouse sperm incubated under NC conditions, in the presence and absence of 1 μg/ml (**A, C, E**) LPS and (**B, D, F**) VLY, where 0 min is the timepoint of bovine serum albumin (BSA) and sodium bicarbonate addition to the sperm suspension. Data are presented as mean and SD (n = 3 biological replicates).
