# Supplementary material for: Bacterial vaginosis toxins impair sperm capacitation and fertilization
Source: Hum Reprod. 2025 Jul 13;40(9):1720–34. doi: 10.1093/humrep/deaf132 (PMC12370371; doi:10.1093/humrep/deaf132)
Supplement: deaf132_Supplementary_Figure_S4 [file deaf132_supplementary_figure_s4.pdf]

Human Reproduction, 2025, 1–1
https://doi.org/10.1093/humrep/deaf132
Supplementary Data

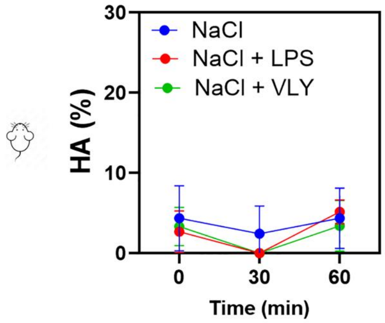

Supplementary Figure S4. The increase in osmolarity by addition of 15 mM sodium chloride into the external medium does not cause lipopolysaccharide (LPS)- and vaginolysin (VLY)-induced mouse sperm hyperactivation. The percentage of sperm with hyperactivated motility (HA) during capacitation, measured by CASA from sperm samples incubated in the presence and absence of 1 μg/ml LPS or VLY, where 0 min is the timepoint when 15 mM sodium chloride (NaCl), was added to the sperm suspension. Data are presented as mean and SD (n = 3 biological replicates). Data were evaluated by two-way ANOVA with Bonferroni's multiple comparison test.
