# Supplementary material for: Bacterial vaginosis toxins impair sperm capacitation and fertilization
Source: Hum Reprod. 2025 Jul 13;40(9):1720–34. doi: 10.1093/humrep/deaf132 (PMC12370371; doi:10.1093/humrep/deaf132)
Supplement: deaf132_Supplementary_Figure_S5 [file deaf132_supplementary_figure_s5.pdf]

OXFORD
human reproduction
Human Reproduction, 2025, 1–1
https://doi.org/10.1093/humrep/deaf132
Supplementary Data

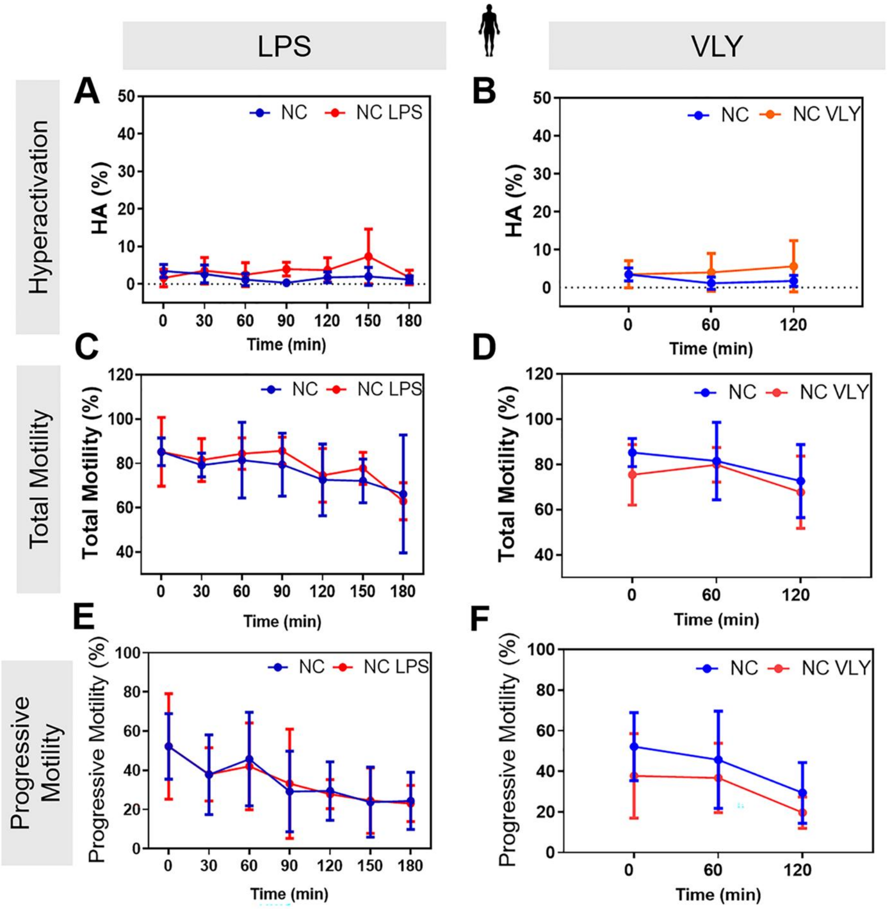

Supplementary Figure S5. Lipopolysaccharide (LPS) and vaginolysin (VLY) do not affect human sperm hyperactivated, total motility, or progressive motility in non-capacitating (NC) conditions. CASA measurements of (**A, B**) hyperactivated (HA), (**C, D**) total motility, and (**E, F**) progressive motility of human sperm in NC conditions in the presence and absence of 0.1 µg/ml (**A, C, E**) LPS and (**B, D, F**) VLY, where 0 min is the timepoint of bovine serum albumin (BSA) and sodium bicarbonate addition to the sperm suspension. Data are presented as mean and SD (n = 5 biological replicates for all experiments).
