# Supplementary material for: Bacterial vaginosis toxins impair sperm capacitation and fertilization
Source: Hum Reprod. 2025 Jul 13;40(9):1720–34. doi: 10.1093/humrep/deaf132 (PMC12370371; doi:10.1093/humrep/deaf132)
Supplement: deaf132_Supplementary_Figure_S6 [file deaf132_supplementary_figure_s6.pdf]

OXFORD
human reproduction
Human Reproduction, 2025, 1–1
https://doi.org/10.1093/humrep/deaf132
Supplementary Data

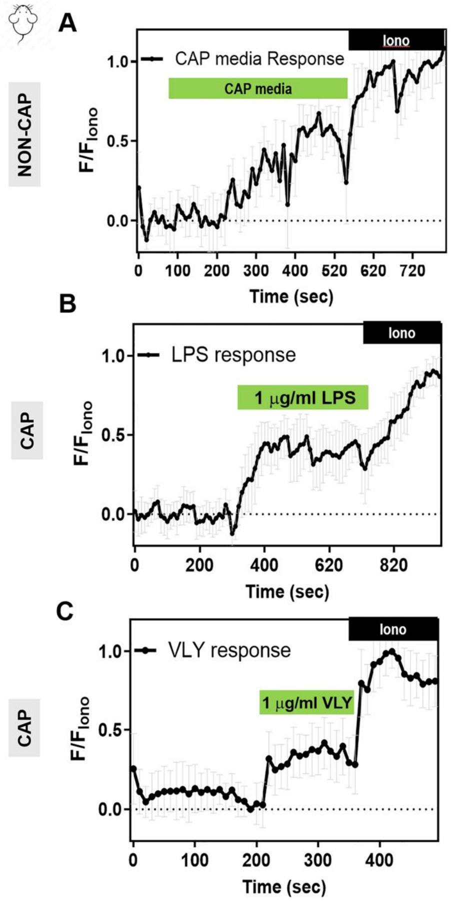

Supplementary Figure S6. Sperm intracellular calcium ($[Ca^{2+}]_i$) response in wild-type (WT) mouse sperm is faster in the presence, than in the absence, of lipopolysaccharide (LPS) or vaginolysin (VLY). Representative traces of $[Ca^{2+}]_i$ response in (**A**) Non-Capacitating (NON-CAP) mouse sperm when exposed to capacitating (CAP) media, followed by 5 µM Ionomycin (Iono). Similar traces were obtained for CAP mouse sperm when exposed to (**B**) LPS and (**C**) VLY. Each trace was normalized to its respective ionomycin (Iono) response. Data are presented as mean and SD (n = 3 biological replicates for **A, B, C**).
