# Supplementary material for: Bacterial vaginosis toxins impair sperm capacitation and fertilization
Source: Hum Reprod. 2025 Jul 13;40(9):1720–34. doi: 10.1093/humrep/deaf132 (PMC12370371; doi:10.1093/humrep/deaf132)
Supplement: deaf132_Supplementary_Figure_S7 [file deaf132_supplementary_figure_s7.pdf]

human reproduction

Human Reproduction, 2025, 1–1
https://doi.org/10.1093/humrep/deaf132
Supplementary Data

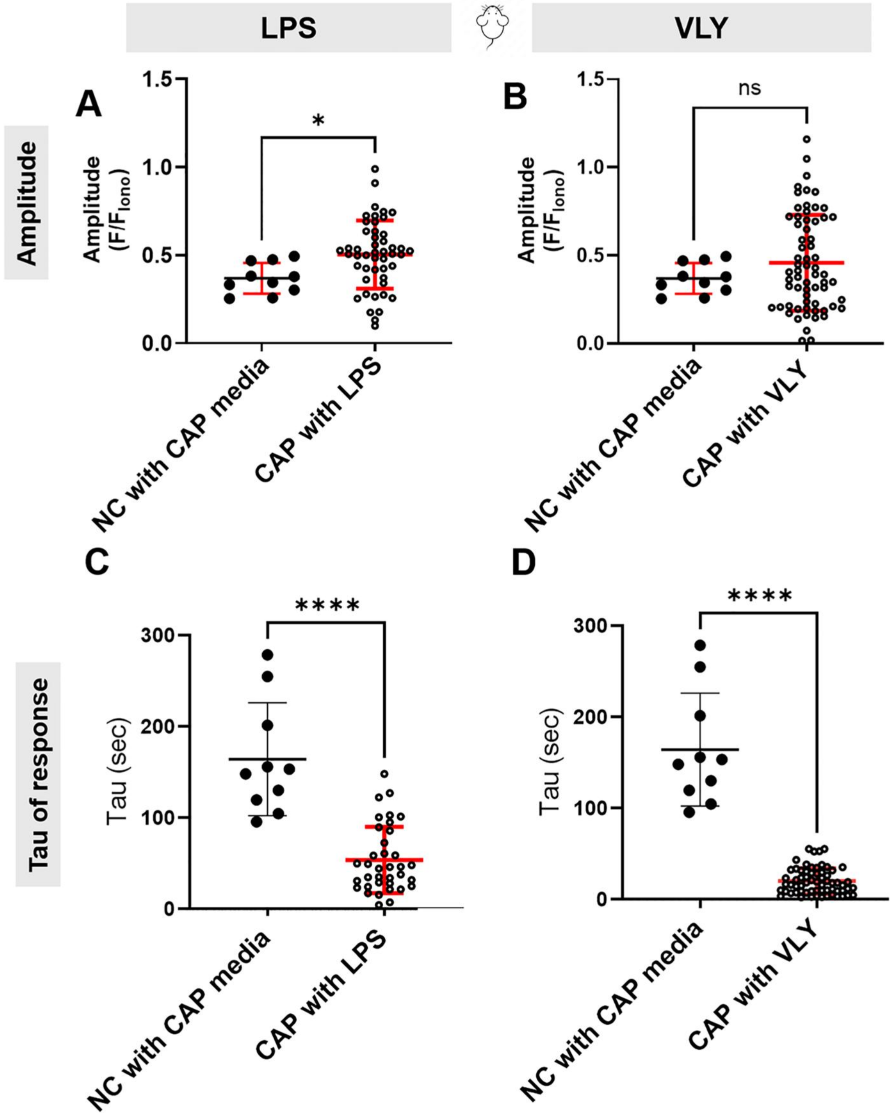

Supplementary Figure S7. Quantified sperm intracellular calcium ([Ca^{2+}]$_i$) response in wild-type (WT) mouse sperm is larger and faster in the presence, than in the absence, of lipopolysaccharide (LPS) or vaginolysin (VLY). (**A, B**) Amplitude and (**C, D**) tau of [Ca^{2+}]$_i$ response in non-capacitating (NC) sperm on acute exposure to (**A, B, C, D**) capacitating (CAP) media, or in CAP sperm on acute exposure to (**A, C**) LPS or (**B, D**) VLY. Data are presented as mean and SD (n = 3 biological replicates for all experiments). *$P < 0.05$, ****$P < 0.001$ by unpaired *t*-test.
