# Supplementary material for: Bacterial vaginosis toxins impair sperm capacitation and fertilization
Source: Hum Reprod. 2025 Jul 13;40(9):1720–34. doi: 10.1093/humrep/deaf132 (PMC12370371; doi:10.1093/humrep/deaf132)
Supplement: deaf132_Supplementary_Figure_S9 [file deaf132_supplementary_figure_s9.pdf]

OXFORD
human reproduction
Human Reproduction, 2025, 1–1
https://doi.org/10.1093/humrep/deaf132
Supplementary Data

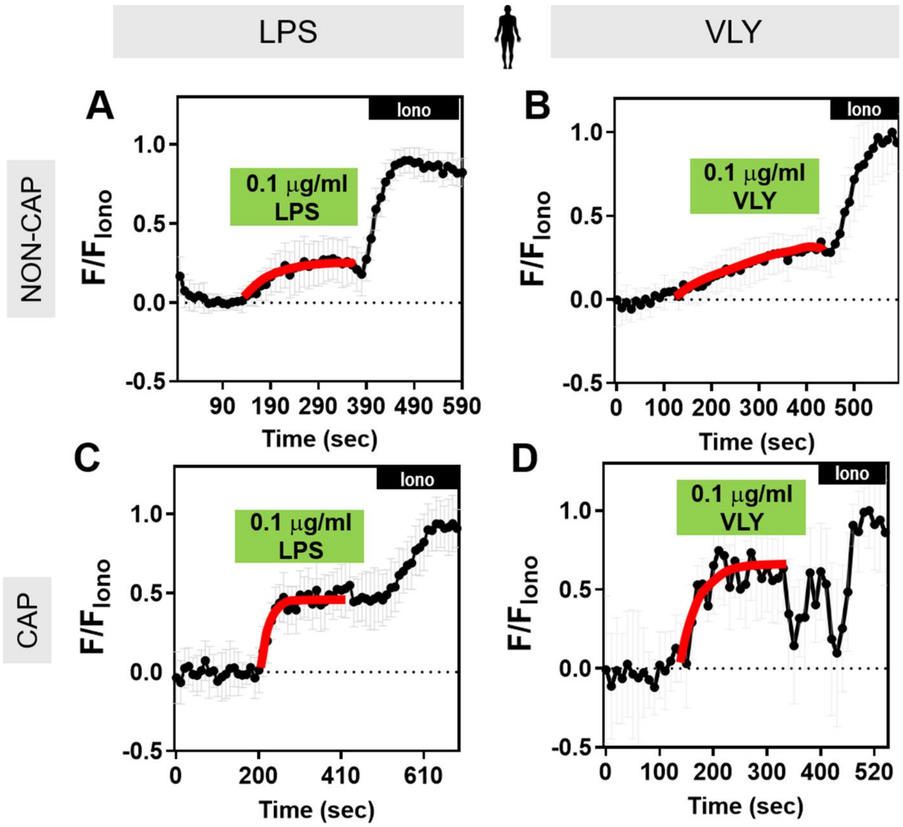

Supplementary Figure S9. Lipopolysaccharide (LPS) and vaginolysin (VLY) at low concentrations induce changes in intracellular calcium ($[Ca^{2+}]_i$) response in human sperm are larger and faster in capacitating (CAP) than in non-capacitating (NC) conditions. Representative traces of (**A, C**) LPS- and (**B, D**) VLY-induced $[Ca^{2+}]_I$ response in (**A, B**) NC and (**C, D**) CAP human sperm. The red curves are standard exponential fits. Each trace was normalized to its respective ionomycin (Iono) response. Data are presented as mean and SD (n = 3 biological replicates for all experiments).
