# Supplementary material for: Bacterial vaginosis toxins impair sperm capacitation and fertilization
Source: Hum Reprod. 2025 Jul 13;40(9):1720–34. doi: 10.1093/humrep/deaf132 (PMC12370371; doi:10.1093/humrep/deaf132)
Supplement: deaf132_Supplementary_Figure_S10 [file deaf132_supplementary_figure_s10.pdf]

Human Reproduction, 2025, 1–1
https://doi.org/10.1093/humrep/deaf132
Supplementary Data

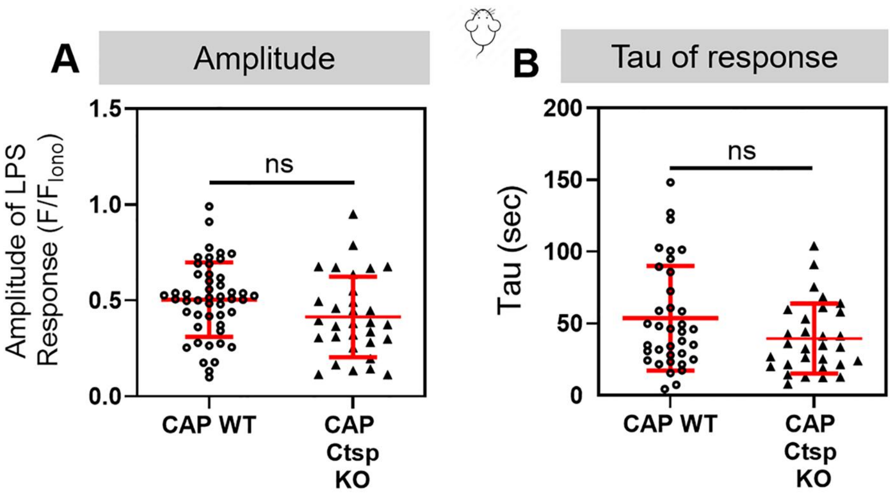

Supplementary Figure S10. Lipopolysaccharide (LPS)-induced intracellular calcium ($[Ca^{2+}]_i$) increases are similar in sperm from wild-type and CatSper knockout mice. (**A**) Amplitude and (**B**) Tau values for the 0.1 µg/ml LPS response in wild-type (WT) and CatSper knockout (KO) sperm in capacitating (CAP) conditions. Data are presented as mean and SD (n = 3 biological replicates for **A, B**). ns, non-significant by unpaired *t*-test.
