# Supplementary material for: Bacterial vaginosis toxins impair sperm capacitation and fertilization
Source: Hum Reprod. 2025 Jul 13;40(9):1720–34. doi: 10.1093/humrep/deaf132 (PMC12370371; doi:10.1093/humrep/deaf132)
Supplement: deaf132_Supplementary_Figure_S11 [file deaf132_supplementary_figure_s11.pdf]

Human Reproduction, 2025, 1–1
https://doi.org/10.1093/humrep/deaf132
Supplementary Data

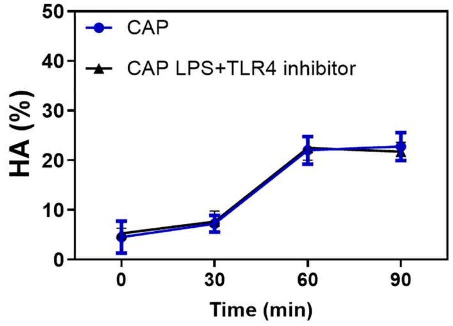

Supplementary Figure S11. Lipopolysaccharide (LPS) effects on mouse sperm hyperactivation are specific and blocked by toll-like receptor 4 (TLR4) inhibitor. CASA measurements were obtained for hyperactivated motility (HA) of mouse sperm incubated under capacitating (CAP) conditions in the presence and absence of 1 μg/ml LPS+10 μM TAK-242 (TLR4 inhibitor). Data are presented as mean and SD (n = 3 biological replicates).
