# Supplementary material for: Bacterial vaginosis toxins impair sperm capacitation and fertilization
Source: Hum Reprod. 2025 Jul 13;40(9):1720–34. doi: 10.1093/humrep/deaf132 (PMC12370371; doi:10.1093/humrep/deaf132)
Supplement: deaf132_Supplementary_Table_S1 [file deaf132_supplementary_table_s1.pdf]

Human Reproduction, 2025, 1–1
https://doi.org/10.1093/humrep/deaf132
Supplementary Data

Supplementary Table S1. Preparation of standards using endotoxin-free water or LAL reagent water.

Standard Conc (EU/ml)	Endotoxin (µl)	Water (µl)
50	50 µl of 1000 EU/ml stock	950
15	15 µl of 1000 EU/ml stock	985
5	5 µl of 1000 EU/ml stock	995
1.5	1.5 µl of 1000 EU/ml stock	998.5
0.5	100 µl of 5 EU/ml stock	900
0.15	30 µl of 5 EU/ml stock	970
0.05	10 µl of 5 EU/ml stock	990
0.015	3 µl of 5 EU/ml stock	997
0.005	1 µl of 5 EU/ml stock	999
0	–	1000

© The Author(s) 2025. Published by Oxford University Press on behalf of European Society of Human Reproduction and Embryology.
This is an Open Access article distributed under the terms of the Creative Commons Attribution License (https://creativecommons.org/licenses/by/4.0/), which permits unrestricted reuse, distribution, and reproduction in any medium, provided the original work is properly cited.
Human Reproduction, 2025, 00, 1–27
https://doi.org/10.1093/humrep/deaf132
Original Article
